# Preosteoclast plays a pathogenic role in syndesmophyte formation of ankylosing spondylitis through the secreted PDGFB — GRB2/ERK/RUNX2 pathway

**DOI:** 10.1186/s13075-023-03142-3

**Published:** 2023-10-05

**Authors:** Yulong Tang, Kai Yang, Qingmei Liu, Yanyun Ma, Hao Zhu, Kunhai Tang, Chengchun Geng, Jiangnan Xie, Dachun Zhuo, Wenyu Wu, Li Jin, Wenze Xiao, Jiucun Wang, Qi Zhu, Jing Liu

**Affiliations:** 1 State Key Laboratory of Genetic Engineering, School of Life Science, and Human Phenome Institute, Zhangjiang Fudan International Innovation Center, Fudan University, Shanghai, China; 2 Department of Dermatology, Jing’an District Central Hospital, Shanghai, China; 3 Division of Dermatology, Huashan Hospital, Fudan University, Shanghai, China; 4 Stem Cell Base, Shanghai East Hospital, Shanghai, China; 5 Department of Rheumatology, Shanghai Pudong Hospital, Fudan University Pudong Medical Center, Shanghai, China; 6 Research Unit of Dissecting the Population Genetics and Developing New Technologies for Treatment and Prevention of Skin Phenotypes and Dermatological Diseases (2019RU058), Chinese Academy of Medical Sciences, Shanghai, China; 7 Institute of Arthritis Research, Shanghai Academy of Chinese Medical Sciences, Guanghua Integrative Medicine Hospital, Shanghai, China; 8 Arthritis Institute of Integrated Traditional and Western Medicine, Shanghai Chinese Medicine Research Institute, Shanghai, China

**Keywords:** Ankylosing spondylitis, Syndesmophyte formation, PDGFB, Osteogenesis

## Abstract

**Objectives:**

Ankylosing spondylitis (AS) is a chronic inflammatory disease that mainly affects the sacroiliac joint and spine. However, the real mechanisms of immune cells acting on syndesmophyte formation in AS are not well identified. We aimed to find the key AS-associated cytokine and assess its pathogenic role in AS.

**Methods:**

A protein array with 1000 cytokines was performed in five AS patients with the first diagnosis and five age- and gender-matched healthy controls to discover the differentially expressed cytokines. The candidate differentially expressed cytokines were further quantified by multiplex protein quantitation (3 AS-associated cytokines and 3 PDGF-pathway cytokines) and ELISA (PDGFB) in independent samples (a total of 140 AS patients vs 140 healthy controls). The effects of PDGFB, the candidate cytokine, were examined by using adipose-derived stem cells (ADSCs) and human fetal osteoblast cell line (hFOB1.19) as in vitro mesenchymal cell and preosteoblast models, respectively. Furthermore, whole-transcriptome sequencing and enrichment of phosphorylated peptides were performed by using cell models to explore the underlying mechanisms of PDGFB. The xCELLigence system was applied to examine the proliferation, chemotaxis, and migration abilities of PDGFB-stimulated or PDGFB-unstimulated cells.

**Results:**

The PDGF pathway was observed to have abnormal expression in the protein array, and PDGFB expression was further found to be up-regulated in 140 Chinese AS patients. Importantly, PDGFB expression was significantly correlated with BASFI (Pearson coefficient/*p* value = 0.62/6.70E − 8) and with the variance of the mSASSS score (mSASSS _2 years − baseline_, Pearson coefficient/*p* value = 0.76/8.75E − 10). In AS patients, preosteoclasts secreted more PDGFB than the healthy controls (*p* value = 1.16E − 2), which could promote ADSCs osteogenesis and enhance collagen synthesis (COLI and COLIII) of osteoblasts (hFOB 1.19). In addition, PDGFB promoted the proliferation, chemotaxis, and migration of ADSCs. Mechanismly, in ADSCs, PDGFB stimulated ERK phosphorylation by upregulating GRB2 expression and then increased the expression of RUNX2 to promote osteoblastogenesis of ADSCs.

**Conclusion:**

PDGFB stimulates the GRB2/ERK/RUNX2 pathway in ADSCs, promotes osteoblastogenesis of ADSCs, and enhances the extracellular matrix of osteoblasts, which may contribute to pathological bone formation in AS.

**Supplementary Information:**

The online version contains supplementary material available at 10.1186/s13075-023-03142-3.

## Key messages

• PDGFB was elevated in plasma in AS patients than in healthy controls.

• Excessive PDGFB was secreted from preosteoclast and could promote osteogenesis of ADSCs by activating the GRB2/p-ERK/RUNX2 pathways.

• PDGFB enhanced the extracellular matrix of osteoblasts and may contribute to syndesmophyte formation.

## Introduction

Ankylosing spondylitis (AS) is a chronic inflammatory disease mainly affecting the sacroiliac joints and spine [1]. Most AS patients have syndesmophyte formation in both the sacroiliac joints and spine, thus causing stiffness. However, the pathogenesis of bone formation remains incompletely understood. In recent years, many studies have investigated the mechanism of syndesmophyte formation in the sacroiliac joints and spine. Several clinical studies have focused on the relationship between inflammation and bone formation but have obtained contradictory results [2–5]. It remains unknown whether inflammation identified by MRI imaging can predict new syndesmophyte formation. Recently, several studies have examined cytokines that are associated with syndesmophyte formation, such as the Wnt signaling pathway inhibitor dickkopf-1 (DKK1) [6, 7]. However, it remains difficult to predict syndesmophyte formation at the initial stage, and an effective target that could reverse or ameliorate the progression of syndesmophyte formation remains elusive.

Previous studies have identified several cytokines that affect new bone formation. In addition to DKK1, the dual blockade of TNF and IL-17A was reported to ameliorate inflammation and structural damage in a rat model of spondyloarthritis [8], suggesting that TNF and IL-17A have pathogenic effects on bone metabolism. Intriguingly, TGF-β could shift the proinflammatory action of TNF on macrophages to osteoclastogenesis [9]. Longitudinal case–control studies have also shown that VEGF, BMP-7, and serum adipokine levels are associated with radiographic spinal progression or serum markers of bone formation in AS patients [10–12]. Cytokine regulation is complex due to its multifaceted effects on different cells and microenvironments, which has led to contradictory results when investigating the expression levels and functions of cytokines [13–18]. The relationship between inflammation and bone formation in AS remains to be elucidated. To this end, we employed cytokine detection methods to deeply understand cytokine dysregulation in AS along with follow-up functional studies and propose that platelet-derived growth factor subunit B (PDGFB) plays a pathogenic role in syndesmophyte formation in AS.

PDGFB has been well studied and has a wide range of functions, especially in the blood vessels. Many studies have demonstrated that PDGFB positively stimulates bone formation both in cultured cells and in mouse models [19, 20]. Additionally, PDGFB has a strong impact on mesenchymal stem cells, such as altering their proliferation and migration [21]. In addition, null PDGFB is lethal in mice due to defects in several organs. Xie et al. constructed a conditional-PDGFB-knockout mouse model using the Cre-loxP approach and found that trabecular bone mass was largely decreased and bone formation was strongly inhibited in mice with osteoclast-specific Pdgfb mutants [22]. Evidence has shown that increased PDGFB secretion by preosteoclast promotes angiogenesis in subchondral bone and then leads to osteoarthritis development [23]. Feng et al. identified PDGFRB as a therapeutic target of ankylosing spondylitis from microarray datasets and found that PDGFRB was upregulated during the osteogenesis of fibroblasts of AS [24]. A recent study also showed that PDGFB accelerated bone mineralization of enthesis cells of AS [25].

In the current study, we aimed to identify common pathogenic cytokines and explore the pathogenic mechanism of candidate cytokines in AS. PDGFB was elevated in AS patients and correlated with syndesmophyte formation. We found that PDGFB promotes the osteoblastogenesis of ADSCs, activates the GRB2/ERK/RUNX2 pathway in ADSCs, and enhances the extracellular matrix of preosteoblasts, which may contribute to pathological bone formation. This previously unrecognized pathogenic mechanism of PDGFB provides the potential for a novel therapeutic target to treat inflammatory spinal damage in AS.

## Methods

### Subjects

Five Chinese AS patients with a first diagnosis and five sex- and age-matched healthy subjects were recruited in the discovery stage. Forty Chinese AS patients and twenty gender- and age-matched healthy patients were included in the validation I stage. The validation II stage included 40 Chinese AS patients who had stopped drug use for at least 1 month, 60 Chinese late-stage AS patients, and 120 healthy Chinese subjects. The 40 AS patients in validation II underwent X-rays of the spine at baseline and at the 2-year follow-up. AS patients with a first diagnosis were those who had received an AS diagnosis but had never been treated according to AS treatment guidelines. AS patients with late-stage disease had a radiological score of the sacroiliac joints of bilateral grade 3–4 and a radiological score of the spine (modified Stoke Ankylosing Spondylitis Spinal Score (mSASSS)) greater than 20, which requires bridging of at least 3 vertebrae or syndesmophytes at the scored vertebral sites [26]. All of the imaging scores were evaluated by two experienced physicians [27]. The diagnosis of AS was performed according to the 1984 modified New York criteria of AS [28]. All participants signed written informed consent forms, and this study was approved by the institutional review boards of Guanghua Integrative Medicine Hospital (2021-K-31) and Shanghai Pudong Hospital (2021-DS-Q-26). The details of the subjects are shown in Table S1.

### Protein array

Protein expression in human plasma was detected by the Human Antibody Array 1000 produced by RayBio (RayBiotech; Cat# AAH-BLG-1000; USA) in the discovery stage. It was performed according to the standard protocol provided by the manufacturer (https://www.raybiotech.com/files/manual/Antibody-Array/AAH-BLG-1000.pdf).

### Multiplex protein quantification and ELISA

Protein quantification of plasma in the validation I stage was performed using ProcartaPlex immunoassays (Invitrogen). We designed the panels (6 cytokines: IL-6, IL-23, IFN-γ, PDGFB, VEGF-D, and VEGF-R) and performed the assays according to the manual provided by the manufacturer. Further quantification was performed on a Luminex 200, and the data analysis was performed according to a standard protocol (https://assets.thermofisher.com/TFS-Assets/LSG/manuals/ProcartaPlex_Analyst_1.0_SW_Manual.June2014.pdf). PDGFB quantification of cultured cells and all patients in the validation II stage was performed using an ELISA kit (Invitrogen, BMS 2071).

### Human spinal entheseal tissues and immunohistochemistry (IHC) analysis

Interlaminar ligamentum flavum, interspinous ligament, or supraspinal ligament tissue was obtained from AS patients and traumatic injury patients who had spinal fractures when undergoing spinal surgery as controls. The tissues were fixed in 4% paraformaldehyde for 4–6 h and then embedded in paraffin. The paraformaldehyde-fixed paraffin-embedded sections were cut into 5 μm using a Leica RM2235. Paraffin-embedded sections were first deparaffinized twice in a series of 100% xylene, rehydrated in a series of graded ethanol (100%, 100%, 95%, and 80%), and then washed briefly in distilled water and PBS, respectively. Antigen thermal repair was performed with 0.01 M citrate buffer (pH6.0) or EDTA buffer (pH9.0) at high pressure, washed in PBS, sections were treated with 3% hydrogen peroxide-methanol for 10 min and blocked with normal goat serum for 30 min. Then, the sections were incubated overnight with PDGFB (1:100, Abcam, catalog: ab23914); GRB2 (1:50, Abcam, catalog: ab32037); P-ERK (1:100, Abcam, catalog: ab278538); RUNX2 (1:50, Abcam, catalog: ab76956) antibody at 4 °C. Goat anti-rabbit IgG secondary antibody (JACKSON, catalog: 111–035-003) was incubated and DAB solution (Sigma, catalog: D8001) was used for color development. All images were obtained using an Open-field slice scanner (NanoZoomer S210).

### Osteoclastogenesis assays

The osteoclastogenesis assays were performed according to our previous study [29]. In brief, human peripheral blood mononuclear cells (PBMCs) were isolated by Ficoll reagent (GE Healthcare, Buckinghamshire, UK) and then monocytes were purified by anti-CD14-conjugated magnetic microbeads (Miltenyibiotech, Bergisch Gladbach, Germany). To induce osteoclast differentiation, monocytes were stimulated with M-CSF (25 ng/mL, R&D Systems, 216-MC-010) and RANKL (40 ng/mL, R&D Systems, 390-TN-010) supplemented with 10% FBS and 10% human serum. Tartrate-resistant acid phosphatase (TRAP) staining was performed with Acid Phosphatase Leucocyte Kit (ZuoChengBio, ZCIC216), and TRAP-positive multinucleated cells (MNC) containing three or more nuclei were counted as preosteoclasts.

### Cell culture and the treatments

Human adipose tissue was obtained through elective liposuction with informed consent. The isolation and expansion of adipose-derived MSCs (ADSCs) was conducted according to previously published techniques [30]. Briefly, lipoaspirate was transferred into 50 mL tubes and centrifuged at 400 × g for 5 min. After digestion with collagenase I and filtration through a 100-μm filter, the stromal vascular fraction (SVF) was obtained. The cells were cultured in 175 cm^2^ flasks until the fifth passage and then used for cell therapy. The ADSCs were characterized by flow cytometry and were found to express CD73, CD90, and CD105 but not express CD31, CD34, CD45, or HLA-DR, and the detector gain optimization and data quality checks were performed according to Gao et al. [31]. The ADSCs were first isolated and cultured in α-modified Eagle’s medium (α-MEM) supplemented with 10% FBS and antibiotics (100 U/mL penicillin and streptomycin) at 37 °C in a 5% CO_2_ humidified incubator for approximately 3 days (coverage > 80%) after which the medium was changed to osteogenic medium (α-MEM plus 50 μM vitamin C, 10 mM β-phosphoglycerol and 100 nM dexamethasone) supplemented with 6% FBS and incubated for 21 days. The hFOB1.19 cells were obtained from the Shanghai Institute of Cell Biology, Chinese Academy of Sciences (Shanghai, China). The hFOB1.19 cells were cultured in Dulbecco’s modified Eagle’s medium/nutrient mixture F-12 (DMEM/F12) supplemented with 10% FBS, 2 mmol/L L-glutamine, 100 U/mL penicillin and streptomycin, 0.3 mg/mL G418, and 1.5 g/L NaHCO3 at 33.5 °C in a 5% CO_2_ humidified incubator and were stimulated with recombinant PDGFB (Abcam, ab259425) for 14 days. For transfection experiments, cells were transfected with 2.5 nmol/ml GRB2 or RUNX2 small interfering (si)RNA mixed with 2 nmol/ml Lipofectamine RNAiMAX transfection reagent (Thermo Fisher Scientific, Waltham, MA, USA). The ERK inhibitor was purchased from Selleck (SCH772984) and was worked in a concentration of 300 nM.

### Alkaline phosphatase (ALP) staining, Alizarin Red S (ARS) staining, and ADSC proliferation, migration, and chemotaxis

ALP staining was performed according to a published article [32]. ARS staining was performed with Alizarin red S kit (ZuoChengBio, ZCIC123) according to the manufacturer’s instructions. The proliferation, migration, and chemotaxis experiments were also performed with the xCELLigence system according to previously published articles [33, 34].

### RNA isolation, cDNA synthesis, real-time RT‒PCR and western blotting

TRIzol reagent was used to extract total RNA from the cells according to the manufacturer’s instructions (Invitrogen). A High Capacity cDNA Reverse Transcription Kit (Applied Biosystems, Foster City, CA, USA) was used to perform reverse transcription according to the manufacturer’s protocol. Real-time PCR samples were mixed with SYBR Premix Ex Taq (TaKaRa Biotech, Tokyo, Japan) and analyzed with an ABI Prism 7900 detector system (Applied Biosystems). β-actin was used as an internal control, and all primers used in this study are shown in Table S2. The assay statistics were analyzed with SDS 2.3 software (Applied Biosystems).

The cellular lysates extracted from cells were used for protein assays. The protein concentration was detected by a spectrophotometer according to the BCA protein assay kit procedure. Equal amounts of protein were subjected to SDS‒PAGE on a 10% polyacrylamide gel and transferred to a polyvinylidene difluoride membrane (Millipore). The membrane with blotted protein was blocked for 1 h at room temperature with a blocking buffer containing 5% BSA and then incubated with antibodies overnight at 4 °C. A housekeeping gene (GAPDH) was used as an internal control. The membrane was then incubated for 1 h at room temperature with secondary horseradish peroxidase-conjugated goat anti-rabbit or anti-mouse IgG after three 10-min washes with TBST. The protein bands were visualized with an ECL solution. Antibodies against ERK1/2 (ab201015), p-ERK1/2 (ab278538), GRB2 (ab32037), RUNX2 (ab76956), ALP (ab83259), type I collagen (ab260043), and type III collagen (ab184993) were purchased from Abcam.

### Whole transcriptome sequencing (RNA sequencing) and pathway enrichment analysis

Total RNA was converted into a DNA library according to the Illumina protocol (TruSeq Stranded Total RNA Sample Preparation Guide). The quality and the intact DNA were examined with an Agilent 2100, and subsequent DNA sequencing was performed with a HiSeq X Ten (Illumina) according to the standard protocol. The RNA sequencing data analysis was performed using TopHat and Cufflink according to a previously published article [35]. Pathway enrichment, including GO and KEGG analysis, was performed with the Functional Interpretation of Differential Expression Analysis (FIDEA) database and KOBAS 3.0 [36, 37].

### Enrichment of phosphorylated peptides using titanium dioxide

Phosphorylation characterization of the peptides was performed according to a previously published article [38]. Briefly, the samples were loaded onto a microcolumn packed with TiO_2_. The phosphopeptides were then eluted from the column. The collected phosphopeptides were then eluted from a microcolumn packed with R3. Finally, the phosphopeptides were analyzed by LC–MS.

### Statistical analysis and plots

All of the experiments were performed in triplicate. The statistical analyses were performed with R software (v3.6.0). Unpaired sample *t*-test was used for unpaired samples and two-way ANOVA was used to evaluate the difference between the two treatments and their effect size. *P* values < 0.05 following FDR adjustment for multiple testing corrections were considered to be statistically significant. All of the plots were generated by ggplot2 in R software.

## Results

### PDGFB was elevated in AS samples both in discovery stage and validation stage compared to healthy controls

**Fig. 1:**
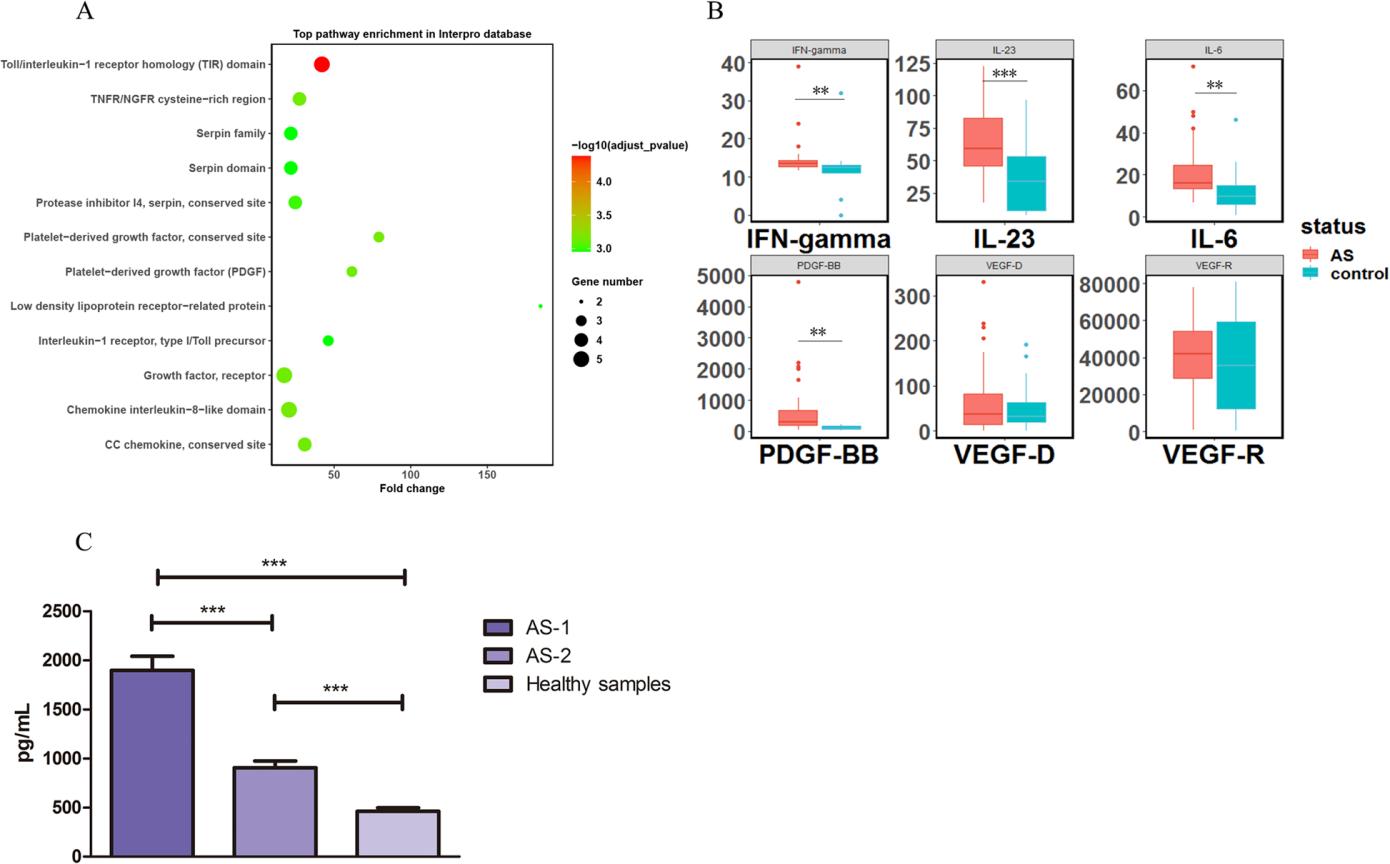
Cytokines detected in AS patients and healthy controls. **A** Top pathway enrichment according to the InterPro database. **B** Differential expression of six cytokines in AS and healthy controls in validation I stage. **C** Differential expression of PDGFB in Chinese AS patients and healthy controls in the validation II stage. AS-1 represents the 40 AS patients at baseline in the validation II stage, and AS-2 represents the 60 late-stage AS patients who have had a disease duration longer than 5 years. The expression levels of cytokines were compared by unpaired sample *t*-tests. *** P < 0.005, ** P < 0.01

A protein array (1000 cytokines) was performed in 5 AS patients with a first diagnosis and 5 healthy samples (Fig. S1A, Table S3). Following the screening approach, several AS-associated pathways, such as Toll receptor homology, CC chemokine, and TNFR, were significantly clustered (Fig. 1A). Interestingly, the vascular-related pathway was also abnormally expressed in AS (Fig. 1A and S1B). We then detected the plasma expression of three vascular-related cytokines along with three known associated cytokines in 40 AS patients and 20 healthy subjects in the validation I stage. The results showed that three known associated cytokines were also significantly elevated in the AS patients. The fold change (FC)/*p* values of IL-6, IL-23, and IFN-γ were 1.75/8E − 3, 1.68/9E − 4, and 1.27/8E − 3, respectively. PDGFB was significantly higher in AS patients (Fig. 1B, FC/*p* value = 5.44/6E − 4). In the validation II stage, we detected the plasma expression of PDGFB in a Chinese cohort (100 AS patients and 120 healthy subjects). PDGFB was consistently more highly expressed in the AS patients (FC/*p* value = 2.67/2.2E − 14). In the Chinese cohort, AS patients with a first diagnosis had much higher PDGFB expression levels than AS patients with a long disease duration (> 5 years) (Fig. 1C).

### PDGFB secreted from preosteoclasts was associated with the Bath Ankylosing Spondylitis Functional Index (BASFI) and mSASSS variation

**Fig. 2:**
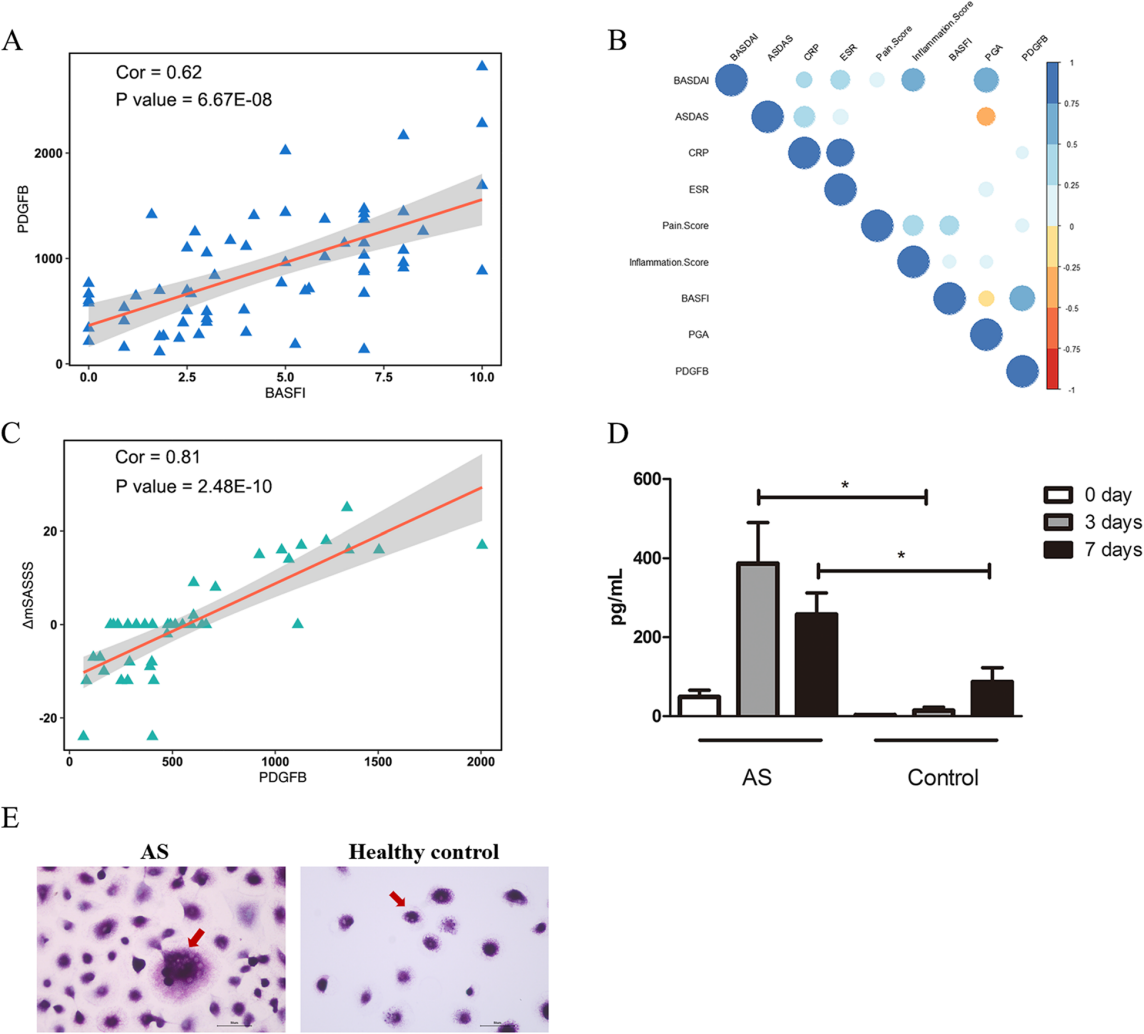
Correlation of PDGFB with clinical indices. **A** Correlation between PDGFB and BASFI. **B** Correlation between PDGFB and all clinical information. **C** Correlation between PDGFB and ΔmSASSS in Chinese AS patients in validation II-40. **D** Expression level of PDGFB secreted from monocytes after stimulation with M-CSF and RANKL for 7 days, which were analyzed by ELISA. **E** TRAP staining of the osteoclastogenesis assay for 7 days stimulation. The expression levels of PDGFB in the AS group and control group were compared by unpaired sample *t*-tests. * P < 0.05

PDGFB expression was positively correlated with the BASFI. The Pearson coefficient was 0.62 (*p* value = 6.7E − 8, Fig. 2A). PDGFB also showed marginal associations with C-reactive protein (CRP) and the pain score (Pearson’s coefficient/*p* value = 0.16/0.02 and 0.20/0.01, respectively), while it showed no association with the disease activity index (BASDAI), patient global assessment (PGA) or AS disease activity score (ASDAS) (Fig. 2B). Interestingly, we found that the plasma expression of PDGFB at baseline was associated with variance in the mSASSS (mSASSS _2 years later − baseline_) in AS patients in validation II-40 (Pearson’s coefficient/*p* value = 0.76/8.75E − 10; Fig. 2C). To explore the origin of the excess PDGFB, monocytes were harvested from AS patients and healthy controls, and osteoclastogenesis assays were performed. Following stimulation for 3 days, the monocytes from AS patients secreted more PDGFB than those of healthy subjects (Fig. 2D, *p* value = 1.16E − 2). The result of the osteoclastogenesis assay was examined by TRAP staining and the monocytes were differentiated into pre-osteoclast and osteoclast 7 days after the stimulation (Fig. 2E).

### PDGFB promoted the osteogenic differentiation, proliferation, chemotaxis, and migration of ADSCs

**Fig. 3:**
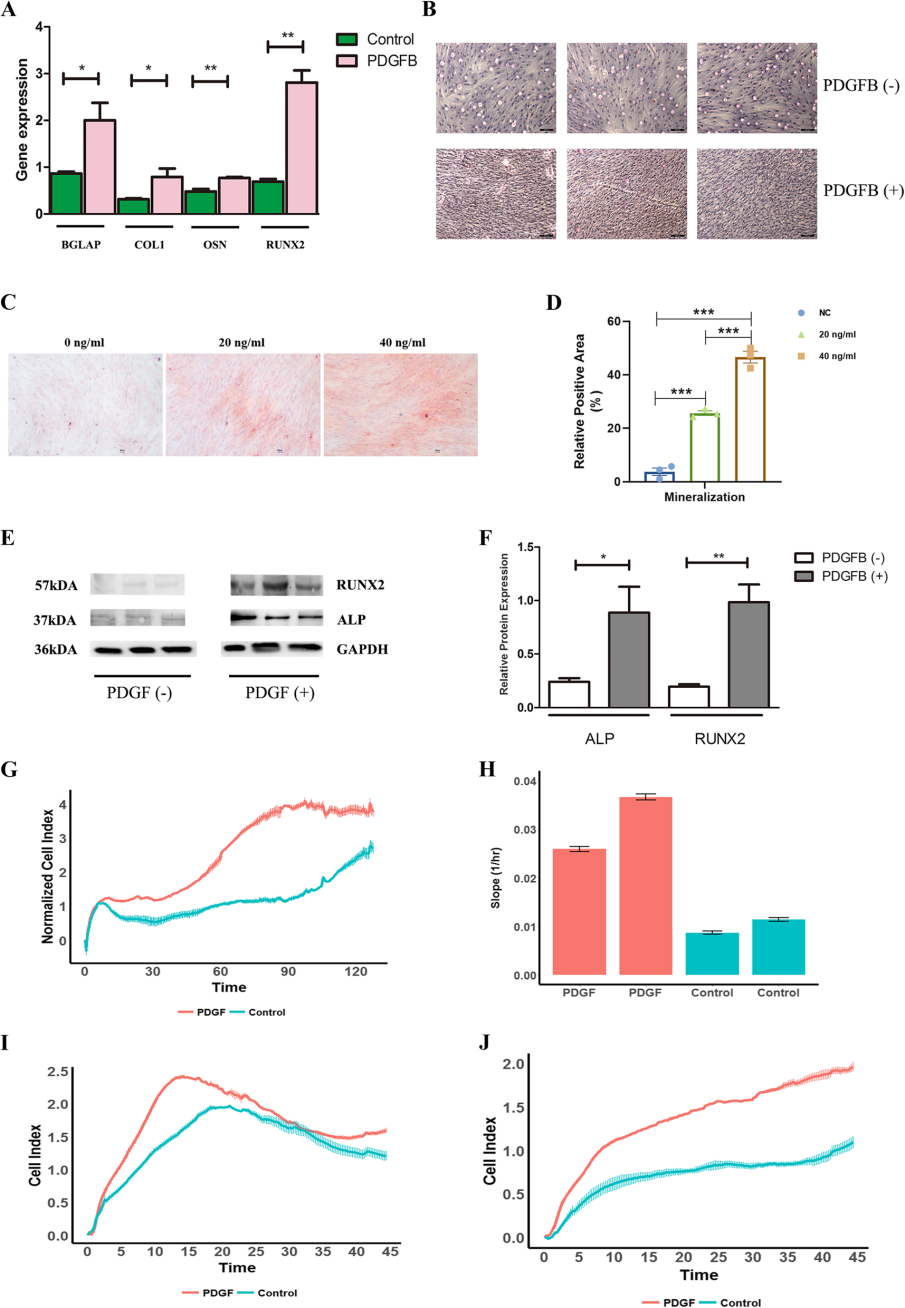
Effects of PDGFB on ADSCs. **A** The mRNA expression of several osteogenic-related genes in the two groups. **B** ALP staining of ADSCs in the two groups. **C**, **D** ARS staining of ADSCs in the two groups. **E** Western blot of ADSCs in the two groups. **F** Relative protein levels of ALP and RUNX2 in ADSCs. **G** Proliferation in the two groups. **H** Slope of proliferation in the two groups. **I** Chemotaxis in the two groups. **J** Migration results for the two groups. The gene expression levels and protein levels were compared by unpaired sample *t*-tests. ** P < 0.01, * P < 0.05

PDGFB promoted the differentiation of ADSCs into osteoblasts. The expression levels of OSN, COL1, and RUNX2 were significantly increased after the ADSCs were stimulated with PDGFB (Fig. 3A). Cell staining also showed that ADSCs stimulated with PDGFB had more intense ALP staining and Alizarin red S staining than controls (Fig. 3B–D). In addition, to exclude the possibility of the unequal number of cells in the two groups, normalization was performed with GAPDH and then the expression values of ALP and RUNX2 were quantified. The results showed that ALP and RUNX2 protein expression was much higher in the PDGFB ( +) group than in the PDGFB ( −) group (FC/*p* value = 3.6/0.05 and 5.2/0.009, respectively, Fig. 3E and F). Cell proliferation and migration activities were examined by the xCELLigence system. As shown in Fig. 3G and H, PDGFB increased the proliferation of ADSCs. In addition, PDGFB recruited ADSCs and increased their migration (Fig. 3I and J).

### PDGFB activated the GRB2/ERK/RUNX2 pathway in ADSCs

**Fig. 4:**
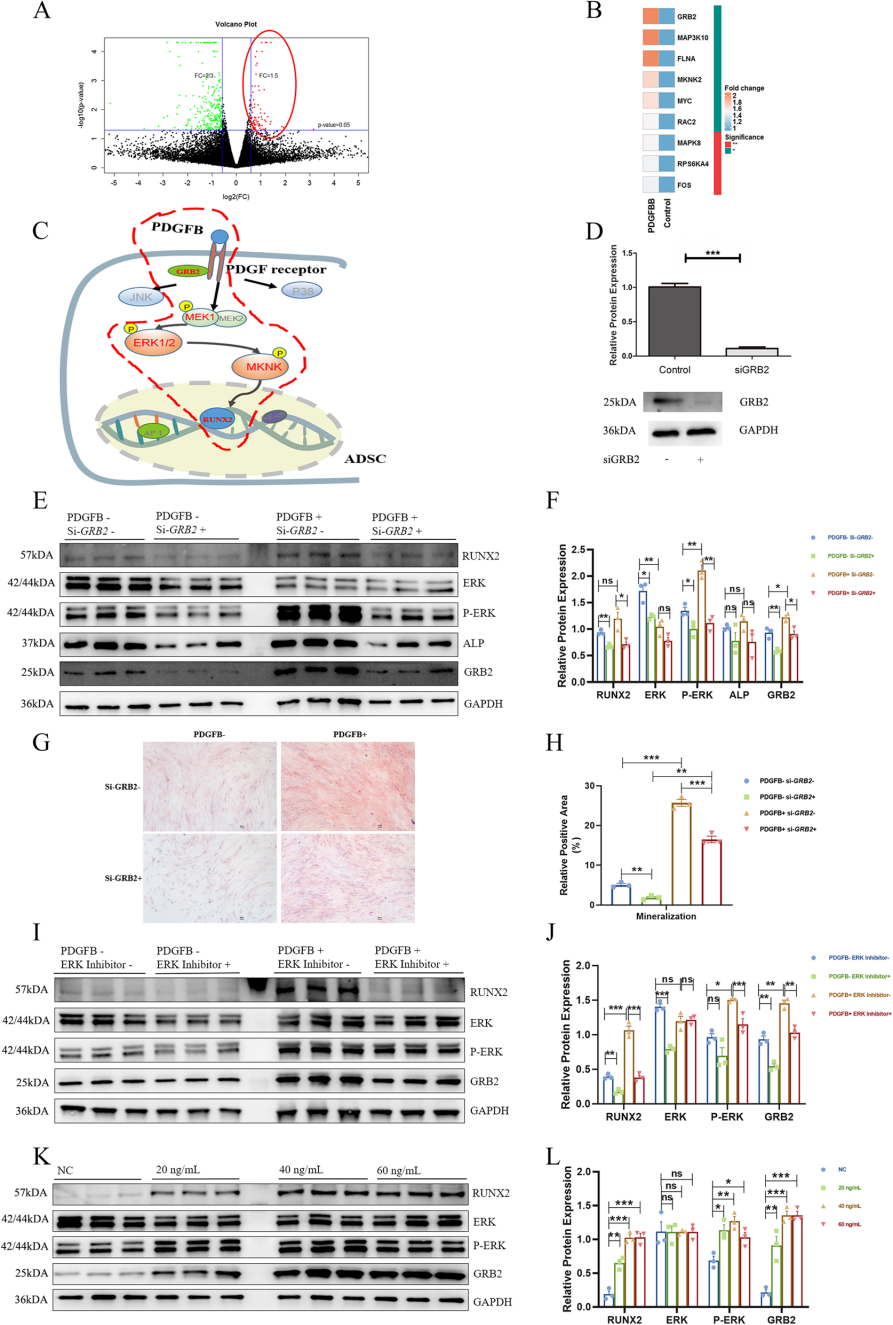
PDGFB pathway regulation in ADSCs. **A** Volcano plot of RNA sequencing in ADSCs for the two groups (PDGF ( −) and PDGF ( +)). **B** Genes with differential expression in the MAPK pathway between the two groups. **C** Differential enrichment of phosphopeptides in ADSCs. MEK1, ERK1/2, and MKNK were identified as differentially phosphorylated in the MAPK pathway. **D** Gene and protein expression of GRB2 after transfection with siGRB2. **E**, **F** Western blot of several key molecules in the GRB2-pERK-RUNX2 axis with/without the treatments of PDGFB and si-*GRB2*. **G**, **H** The mineralization ability of ADSCs with/without the treatments of PDGFB and si-*GRB2*. **I**, **J** Western blot of several key molecules in the GRB2-pERK-RUNX2 axis with/without the treatments of PDGFB and ERK inhibitor*.* **K**, **L** PDGFB activated the GRB2-pERK-RUNX2 axis in a dose-dependent manner. The gene and protein levels were compared by unpaired sample *t*-tests. *** P < 0.005, ** P < 0.01, * P < 0.05

**Fig. 5:**
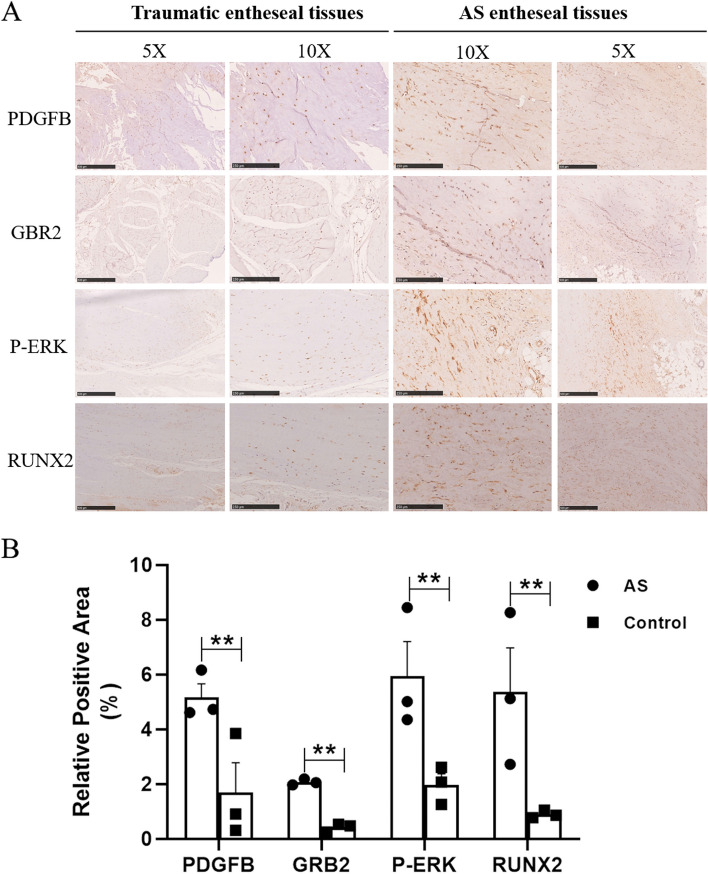
Immunochemistry analysis of the spinal entheseal tissues of AS patients and traumatic injury patients. **A** Elevated expression of the PDGFB, GRB2, P-ERK, and RUNX2 in AS patients. **B** Relative positive area of target protein in the sections were quantified

The results of the whole-transcriptome analysis showed that PDGFB activated the MAPK pathway to strengthen the osteogenic differentiation of ADSCs (Fig. 4A, Table S4). In particular, the expression of GRB2 was significantly increased following stimulation (FC/*p* value = 2.1/0.008) (Fig. 4B). To determine the key phosphorylated molecule in the MAPK pathway, highly selective phosphorylated peptides were analyzed by titanium dioxide chromatography. ERK, MEK1, and MKNK were abnormally phosphorylated after stimulation with PDGFB (Fig. 4C). By considering both the RNA sequencing results and peptide phosphorylation data, we determined that PDGFB stimulates ERK phosphorylation and enhances RUNX2 expression through GRB2. The expression of GRB2 is then inhibited by siRNA in the ADSCs (Fig. 4D). The western blot results showed that in the si-*GRB2* group, ERK phosphorylation and RUNX2 expression were decreased, and the enhancement of GRB2/p-ERK/RUNX2 triggered by PDGFB was also inhibited by silencing the expression of *GRB2* (Fig. 4E and F, Table S5). The ARS staining also showed that si-*GRB2* inhibited the mineralization of ADSCs, and the enhancement of mineralization triggered by PDGFB was also decreased by the treatment of si-*GRB2* (Fig. 4G and H, Table S6). To further explore the effects of the GRB2/p-ERK/RUNX2 pathway triggered by PDGFB, we inhibited the expression of ERK by using the ERK inhibitor. The results showed that when the ADSCs were treated with both PDGFB and ERK inhibitor, the axis was significantly inhibited by ERK inhibition compared with that after stimulation with PDGFB alone (Fig. 4I and J, Table S7, mRNA levels were shown in Fig. S2A). In addition, when the ADSCs were stimulated with PDGFB, the GRB2/p-ERK/RUNX2 axis was activated in dose- (Fig. 4K and L, mRNA levels were shown in Fig. S2B) and time- (Fig. S2C and D, mRNA levels were shown in Fig. S2E) dependent manner, which strengthen the role of PDGFB to this axis. Furthermore, the enhancement of PDGFB and the activation of the GRB2/p-ERK/RUNX2 pathway was found in the spinal entheseal tissues of AS patients (Fig. 5A and B).

### PDGFB Enhanced the Extracellular Matrix (ECM) of preosteoblasts

**Fig. 6:**
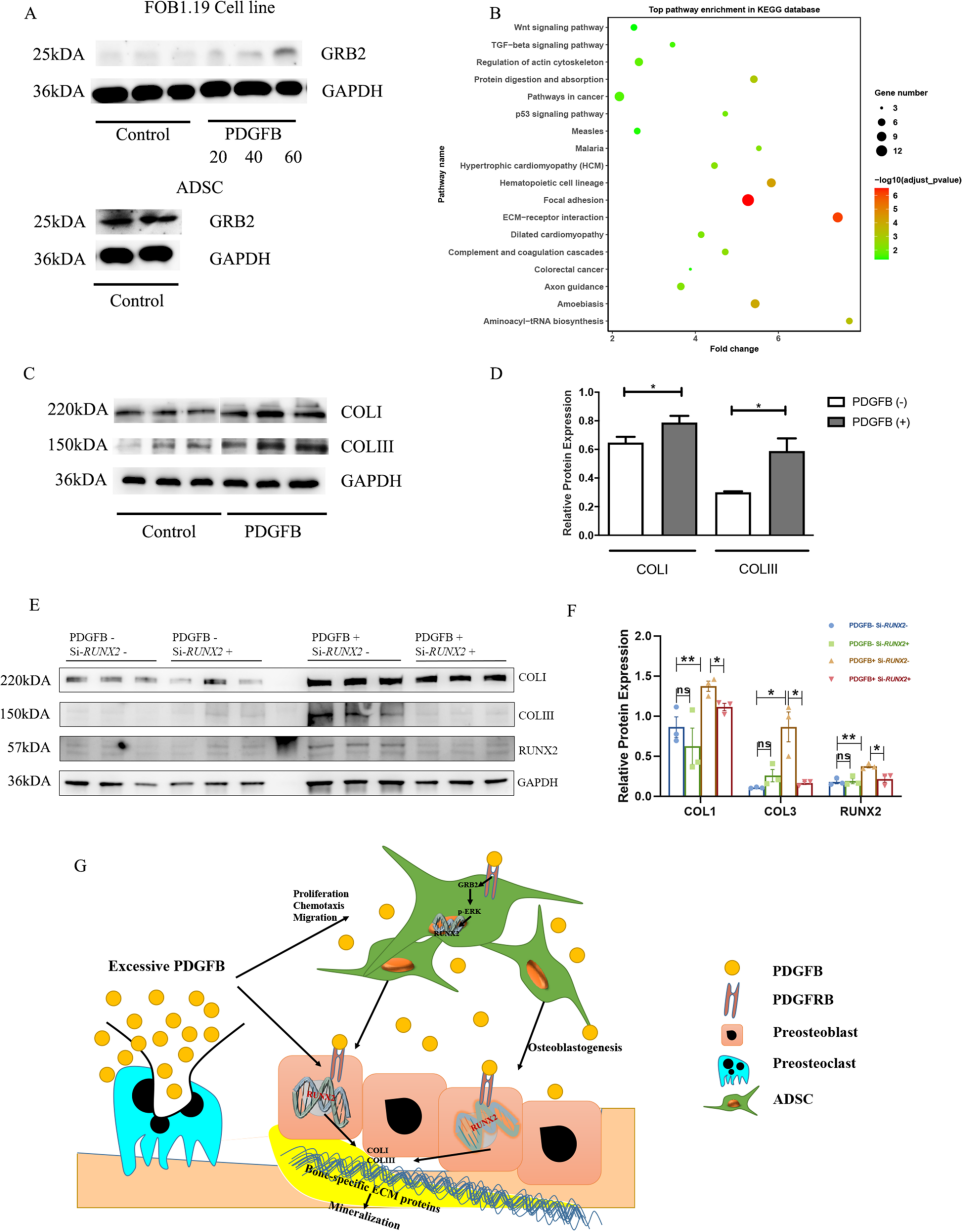
Osteoblastogenic effects of PDGFB. **A** Protein expression of GRB2 in FOB1.19 cells and ADSCs. Western blotting was performed under the same conditions (equal exposure time and dose). **B** Top pathway enrichment in the KEGG database in FOB1.19 cells. **C** Collagen expression of FOB1.19 in the two groups (PDGFB − , PDGFB +). **D** Relative protein level of collagen expression in FOB1.19 in the two groups (PDGFB − , PDGFB +). **E**, **F** Western blot of the collagen expression and RUNX2 with/without the treatments of PDGFB and si-*RUNX2*. **G** Possible mechanism of PDGFB pathogenesis in AS. The relative protein levels were compared by unpaired sample *t*-tests. * P < 0.05

FOB1.19 cells had no response to PDGFB regarding further osteogenic differentiation, proliferation, chemotaxis, or migration (Fig. S3A and B). First, we detected the expression of the axis pathway (GRB2/p-ERK/RUNX2) and found that GRB2 protein expression was relatively low in FOB1.19 cells even after stimulation with 60 ng/mL PDGFB; thus, the MAPK pathway could not be activated after stimulation with PDGFB (Fig. 6A). To further explore the impact of PDGFB on osteoblasts, RNA sequencing was performed. The pathways of focal adhesion and ECM-receptor interaction were significantly enriched according to the KEGG analysis (Fig. 6B and S4B). The detailed genes are presented in Fig. S4C and D, and the fully enriched pathways are presented in Fig. S4A, B and Tables S8 and S9 for both GO and KEGG. The western blot results also showed that the expression of type I and III collagen was significantly enhanced after stimulation with PDGFB (Fig. 6C and D). In addition, to explore whether the enhancement of type I and III collagen was RUNX2 dependent, we silenced the expression of RUNX2 by siRNA. The results showed that the enhancement of COL1 and COL3 triggered by PDGFB were both decreased after silencing the expression of RUNX2 (Fig. 6E and F, Table S10, mRNA levels were shown in Fig. S5), suggesting that the overexpression of COL1 and COL3 by PDGFB stimulation was RUNX2-pathway dependent.

## Discussion

Ankylosing spondylitis (AS) is an immune disease mainly affecting sacroiliac joints and the spine. However, the mechanism of abnormal bone formation in axial joints remains unknown [39]. Many studies have suggested that inflammation has a complex relationship with bone formation [15, 40, 41], but the details of the mechanism are still not well understood. In this study, we aimed to identify an inflammatory cytokine that impacts bone homeostasis and that partly explains ectopic bone formation in AS.

We found that the plasma expression of PDGFB was higher in AS patients than in controls. Furthermore, PDGF expression in AS patients with a first diagnosis was about twice as high as that in AS patients with a long duration of illness. Because the patients with a first diagnosis had not been treated with any drugs, the identified elevated PDGFB expression might be involved in AS pathogenesis in the early stage of the disease. In addition, we found that PDGFB expression was correlated with the formation of syndesmophytes, as evaluated by X-ray. Therefore, we hypothesize that PDGFB is regulated by inflammatory cells and increases the risk of bone formation in AS patients. To demonstrate that abnormal PDGFB was secreted by inflammatory cells, we obtained monocytes from AS patients and healthy people and stimulated the cells with M-CSF and RANKL. The results showed that after being stimulated for 3 days, PDGFB secretion from monocytes peaked and was significantly higher than that of the controls. This result suggested that the excessive PDGFB in AS likely arises from monocytes.

Previous studies have reported that PDGFB promotes osteogenic function, and several studies with mouse models have also demonstrated this [42–44]. Recent studies have also shown that PDGFB can enhance osteogenic capabilities during the use of some alloplastic matrices and when using some gel membrane periosteal models for bone regeneration [45–47]. PDGFB is considered a potent initiator of bone formation [48]. However, some studies have come to the opposite conclusion [49]. The conflicting results suggest that PDGFB might play a role in only some specific cells. Therefore, both the osteoblast cell line and mesenchymal cells were selected for use in this study. The results showed that PDGFB indeed played a role in ADSCs but not in FOB1.19 cells. To further elucidate the underlying mechanism, RNA sequencing was performed. The results showed that the MAPK pathway (p-ERK) was activated in ADSCs by increasing the expression of GRB2. As in the hFOB1.19 cells, PDGFB did not increase ALP protein expression and did not promote cell proliferation. We postulate that p-ERK is not activated by PDGFB in FOB1.19 cells, as p-ERK is well known to promote cell proliferation. The results also showed that the protein expression level of p-ERK remained unchanged after stimulation (data not shown). Interestingly, GRB2 protein expression was very low in FOB1.19 cells, but it slightly increased when the PDGFB concentration reached 60 ng/mL (Fig. S4C). Considering the above results, we suggest that as there was not sufficient GRB2 to bind to the SH2 domain of the PDGF receptor, PDGFB did not have an obvious impact on osteogenesis in osteoblasts. However, by performing RNA sequencing in FOB cells, we found that PDGFB promoted the ECM pathway and the expression of collagen II and collagen III (Fig. S4B). It is well known that the overexpression of collagen-related genes has a pathogenic role. Therefore, PDGFB might play a role in another aspect of osteoblasts.

For at least a decade, it has been widely reported that in AS patients, excessive fat metaplasia exists in the sacroiliac joints and spine [50, 51]. However, we do not know where these fats come from. The results of this study indicate that these fats might have a pathogenic role in bone formation. It has been demonstrated that PDGFB strongly promotes the chemotaxis and migration of ADSCs. Excessive PDGFB from preosteoclasts might attract more ADSCs from fat to the sacroiliac joints and spine and promote the differentiation of ADSCs into preosteoblasts. PDGFB could then enhance ECM/collagen expression in preosteoblasts and accelerate bone formation (Fig. 6G). Therefore, targeting ADSCs and PDGFB might have lessened AS.

### Limitations

Several limitations of this study need to be addressed. Firstly, the specific mechanisms of how PDGFB triggered the GRB2-pERK-RUNX2 pathway were not completely identified. GRB2 was found to contain an SH2 domain which is flanked by two SH3 domains [52, 53]. The SH2 domain recognizes the phosphotyrosine residues of activated EGFR, and the two SH3 domains bind to proline-rich sequences. Therefore, GRB2 can link EGFR and downstream signaling molecules (i.e., pERK), and may also link to PDGF [54]. In addition, the phosphorylation of ERK was reported to regulate skeletal development and bone formation [55]. As pERK is essential for bone formation, the interaction between GRB2 and pERK and how they interacted with RUNX2 were not fully discovered. The activation of the GRB2-pERK-RUNX2 axis triggered by PDGFB warranted deep exploration. Second, the enhancement of the GRB2/p-ERK/RUNX2 axis triggered by PDGFB was examined by normal human ADSCs, and the enhancement or the enhanced osteogenesis in ADSCs from AS patients was not identified and compared. However, Sungsin Jo et al. [25] have demonstrated that after PDGFB stimulation, the mineralization ability of enthesis cells from AS patients was stronger than the healthy ones. In addition, although PDGFB affects the axis in ADSCs, it does not have such effects on BMSCs (data not shown), suggesting its role in bone formation is limited to specific cell types and could not fully explain the mechanisms of new bone formation in AS.

## Conclusions

In conclusion, PDGFB showed higher expression in AS patients and was correlated with the variation in syndesmophyte formation. PDGFB promoted the osteoblastogenesis of ADSCs by activating the GRB2/ERK/RUNX2 pathway and enhanced the extracellular matrix of preosteoblasts, which could contribute to heterotopic bone formation in AS patients. Targeting ADSCs and PDGFB may be a potential therapeutic strategy to ameliorate AS.

### Supplementary Information

**Additional file 1: Table S1.** Demographic characteristics of AS patients in this study.

**Additional file 2: Table S2.** Primer sequences of the target gene.

**Additional file 3: Table S3.** Protein array data for the discovery samples.

**Additional file 4: Table S4.** RNA sequencing data for ADSCs treated with/without PDGFB.

**Additional file 5: Table S5.** Results of western blotting of si-GRB2 and PDGFB treatments analysed by two-way ANOVA.

**Additional file 6: Table S6.** Results of ARS staining of si-GRB2 and PDGFB treatments analysed by two-way ANOVA.

**Additional file 7: Table S7.** Results of western blotting of ERK inhibitor and PDGFB treatments analysed by two-way ANOVA.

**Additional file 8: Table S8.** Pathway enrichment of FOB cells according to GO (Biological Process).

**Additional file 9: Table S9.** Pathway enrichment of FOB cells according to KEGG.

**Additional file 10: Table S10.** Results of western blotting of si-RUNX2 and PDGFB treatments analysed by two-way ANOVA.

**Additional file 11: Figure S1.** A, Heatmap plot of AS and controls. B, PDGFB expression in AS patients and controls. The relative protein levels were compared by unpaired sample t-tests. * P < 0.05.

**Additional file 12: Figure S2.** PDGFB pathway regulation in ADSCs. A, The mRNA expression of *GRB2* and *RUNX2* with/without the treatments of PDGFB and ERK inhibitor. B, The mRNA expression of several key genes in GRB2-pERK-RUNX2 axis and osteogenesis-related genes, in dose-dependent groups. C-D, Western blot of several key molecules in the axis with the treatment of PDGFB in time-dependent groups. E, The mRNA expression of several key genes in the axis and osteogenesis-related genes, in time-dependent groups.

**Additional file 13: Figure S3.** A, The mRNA expression of osteogenic-related genes in FOB1.19 cells. B, ALP staining of FOB1.19 in the two groups. The gene expression levels were compared by unpaired sample t-tests. ** P < 0.01.

**Additional file 14: Figure S4.** A, GO enrichment analysis of RNA sequencing in FOB1.19 cells. B, KEGG enrichment analysis of RNA sequencing in FOB1.19 cells. C, Enriched AGE-RAGE pathway in FOB1.19 cells. D, Enriched ECM-receptor interaction pathway in FOB1.19 cells.

**Additional file 15: Figure S5.** The mRNA expression of several key genes in the GRB2-pERK-RUNX2 axis and several osteogenesis related genes, with/without the treatments of PDGFB and si-*RUNX2*.

## Data Availability

The data and the code that support the findings of this study are available on reasonable request from the corresponding authors.

## Abbreviations

AS: Ankylosing spondylitis
ASDAS: Ankylosing Spondylitis Disease Activity Score
BASDAI: Bath Ankylosing Spondylitis Disease Activity Index
BASFI: Bath Ankylosing Spondylitis Functional Index
ECM: Extracellular matrix
hFOB: Human fetal osteoblast
ADSC: Adipose-derived stem cell
LC-MS: Liquid chromatography-mass spectrometry
PGA: Patient global assessment
BMSC: Bone marrow stem cell
mSASSS: Modified Stoke Ankylosing Spondylitis Spinal Score
PDGFB: Platelet-derived growth factor subunit B
DKK1: Dickkopf-1
GO: Gene ontology
KEGG: Kyoto Encyclopedia of Genes and Genomes
RANKL: Receptor activator of nuclear kappa-B ligand
M-CSF: Macrophage colony-stimulating factor
FDR: False discovery rate
RUNX2: Runt-related transcription factor 2
siRNA: Small interfering RNA
FC: Fold change
CRP: C-reactive protein
NSAIDs: Non-steroid anti-inflammatory drugs
DMARDs: Disease-modifying anti-rheumatic drugs
GRB2: Growth factor receptor-bound protein 2
ERK: Extracellular signal-regulated kinase
ESR: Erythrocyte sedimentation rate
ALP: Alkaline phosphatase
HLA: Human leukocyte antigen
OSN: Osteonectin
TRAP: Tartrate-resistant acid phosphatase
ARS: Alizarin red S
