# Supplementary material for: Preosteoclast plays a pathogenic role in syndesmophyte formation of ankylosing spondylitis through the secreted PDGFB — GRB2/ERK/RUNX2 pathway
Source: Arthritis Res Ther. 2023 Oct 5;25:194. doi: 10.1186/s13075-023-03142-3 (PMC10552372; doi:10.1186/s13075-023-03142-3)
Supplement: Supplementary file 1 — Additional file 1: Table S1. Demographic characteristics of AS patients in this study. [file 13075_2023_3142_MOESM1_ESM.docx]

Table S1 Demographic characteristics of AS patients in this study.

| Parameter | Discovery  (n = 5) | Validation I (n = 40) | Validation II-baseline (n = 40) | Validation II-two years later (n = 40) | Validation II  (n = 60) |
| --- | --- | --- | --- | --- | --- |
| Age, years (SD) | 30.24（18.65） | 36.57 (12.78) | 30.24（18.65） | 32.24（18.65） | 47.12（14.66） |
| Male: Female | 4:1 | 30:10 | 35:5 | 35:5 | 45:15 |
| HLA-B27 (%) | 100 | 100 | 100 | 100 | 100 |
| ESR (SD) | 16.80（7.2） | 23.80 (25.90) | 24.03（27.78） | 6.55（8.29） | 15.31（12.84） |
| CRP (SD) | 12.12（3.4） | 18.41 (25.98) | 13.72（12.98） | 2.77（4.14） | 6.41（9.77） |
| BASDAI (SD) | 4.50（0.89） | 5.94 (1.32) | 6.52（1.57） | 1.21（0.48） | 2.62（2.43） |
| BASFI (SD) | 0.87（1.05） | 3.90 (2.51) | 2.11（1.81） | 0.89（1.11） | 1.33（1.50） |
| ASDAS (SD) | 2.23（0.51） | 3.58 (0.77) | 3.37（0.78） | 1.14（0.69） | 1.78（0.91） |
| mSASSS (SD) | / | / | 13.39 (14.32) | 13.65 (17.12) | >20 |
| Drug treatments (%) | 0 | 91 | /* | 100 | 98 |
| NSAIDs/DMARDs (%) | 0 | 90 | /* | / | 95 |
| TNF blocker (%) | 0 | 56 | /* | 100 | 72 |

Data are presented as the mean (standard deviation) or percentage. HLA: human leucocyte antigen, BASDAI: Bath Ankylosing Spondylitis Disease Activity Index, BASFI: Bath Ankylosing Spondylitis Functional Index, ESR: erythrocyte sedimentation rate, CRP: C-reactive protein, ASDAS: ankylosing spondylitis disease activity score. Drug treatments included NSAIDs, DMARDs, immunosuppressive drugs and TNF blockers. *Patients in validation II-baseline had stopped any drug use for at least one month.
