# Supplementary material for: Preosteoclast plays a pathogenic role in syndesmophyte formation of ankylosing spondylitis through the secreted PDGFB — GRB2/ERK/RUNX2 pathway
Source: Arthritis Res Ther. 2023 Oct 5;25:194. doi: 10.1186/s13075-023-03142-3 (PMC10552372; doi:10.1186/s13075-023-03142-3)
Supplement: Supplementary file 2 — Additional file 2: Table S2. Primer sequences of the target gene. [file 13075_2023_3142_MOESM2_ESM.docx]

Table S2 Primer sequences of the target gene.

| Gene | Forward | Reverse |
| --- | --- | --- |
| *ALP* | GCTGTAAGGACATCGCCTACCA | CCTGGCTTTCTCGTCACTCTCA |
| *COL1* | GAGAGGAAGGAAAGCGAGGAG | GGGACCAGCAACACCATCT |
| *COL3* | TGGTCTGCAAGGAATGCCTGGA | TCTTTCCCTGGGACACCATCAG |
| *GRB2* | GAAATGCTTAGCAAACAGCGGCA | TCCATCTCGGAGCACCTTGAAG |
| *RUNX2* | CCACCGAGACCAACAGAGTC | GTCACTGTGCTGAAGAGGCT |
| *ERK1* | TACCAGTTGCGGTTCACCGTGT | GCAGAGACTGTAGGTAGTTTCGG |
| *ERK2* | ACACCAACCTCTCGTACATCGG | TGGCAGTAGGTCTGGTGCTCAA |
| *OSN* | TCGGCATCAAGCAGAAGGATA | CCAGGCAGAACAACAAACCAT |
| *BGLAP* | TGAGAGCCCTCACACTCCTC | ACCTTTGCTGGACTCTGCAC |
| β-actin | AGCACAGAGCCTCGCCTTT | CCCACGATGGAGGGGAAGA |
