# Supplementary material for: Preosteoclast plays a pathogenic role in syndesmophyte formation of ankylosing spondylitis through the secreted PDGFB — GRB2/ERK/RUNX2 pathway
Source: Arthritis Res Ther. 2023 Oct 5;25:194. doi: 10.1186/s13075-023-03142-3 (PMC10552372; doi:10.1186/s13075-023-03142-3)
Supplement: Supplementary file 5 — Additional file 5: Table S5. Results of western blotting of si-GRB2 and PDGFB treatments analysed by two-way ANOVA. [file 13075_2023_3142_MOESM5_ESM.docx]

Table S5 Results of western blotting of si-GRB2 and PDGFB treatments analysed by two-way ANOVA.

|  | Effect factors | SS | DF | MS | F (DFn, DFd) | P value | P value summary |
| --- | --- | --- | --- | --- | --- | --- | --- |
| RUNX2 | Si-GRB2+PDGFB | 0.03859 | 1 | 0.03859 | F (1, 8) = 2.728 | P=0.1372 | ns |
|  | Si-GRB2 | 0.4128 | 1 | 0.4128 | F (1, 8) = 29.18 | P=0.0006 | *** |
|  | PDGFB | 0.06292 | 1 | 0.06292 | F (1, 8) = 4.447 | P=0.0680 | ns |
| ERK | Si-GRB2+PDGFB | 0.03756 | 1 | 0.03756 | F (1, 8) = 1.993 | P=0.1957 | ns |
|  | Si-GRB2 | 0.4278 | 1 | 0.4278 | F (1, 8) = 22.71 | P=0.0014 | ** |
|  | PDGFB | 0.948 | 1 | 0.948 | F (1, 8) = 50.31 | P=0.0001 | *** |
| P-ERK | Si-GRB2+PDGFB | 0.3249 | 1 | 0.3249 | F (1, 8) = 15.80 | P=0.0041 | ** |
|  | Si-GRB2 | 1.342 | 1 | 1.342 | F (1, 8) = 65.24 | P<0.0001 | **** |
|  | PDGFB | 0.5727 | 1 | 0.5727 | F (1, 8) = 27.85 | P=0.0007 | *** |
| ALP | Si-GRB2+PDGFB | 0.01298 | 1 | 0.01298 | F (1, 8) = 0.2923 | P=0.6035 | ns |
|  | Si-GRB2 | 0.3038 | 1 | 0.3038 | F (1, 8) = 6.842 | P=0.0309 | * |
|  | PDGFB | 0.00761 | 1 | 0.00761 | F (1, 8) = 0.1713 | P=0.6898 | ns |
| GRB2 | Si-GRB2+PDGFB | 0.00058 | 1 | 0.00058 | F (1, 8) = 0.08176 | P=0.7822 | ns |
|  | Si-GRB2 | 0.3265 | 1 | 0.3265 | F (1, 8) = 45.75 | P=0.0001 | *** |
|  | PDGFB | 0.2748 | 1 | 0.2748 | F (1, 8) = 38.50 | P=0.0003 | *** |

Notes: SS, the sum of squares; DF, degree of freedom; MS, mean square.
