# Supplementary material for: Preosteoclast plays a pathogenic role in syndesmophyte formation of ankylosing spondylitis through the secreted PDGFB — GRB2/ERK/RUNX2 pathway
Source: Arthritis Res Ther. 2023 Oct 5;25:194. doi: 10.1186/s13075-023-03142-3 (PMC10552372; doi:10.1186/s13075-023-03142-3)
Supplement: Supplementary file 6 — Additional file 6: Table S6. Results of ARS staining of si-GRB2 and PDGFB treatments analysed by two-way ANOVA. [file 13075_2023_3142_MOESM6_ESM.docx]

Table S6 Results of ARS staining of si-GRB2 and PDGFB treatments analysed by two-way ANOVA.

|  | Effect factors | SS | DF | MS | F (DFn, DFd) | P value | P value summary |
| --- | --- | --- | --- | --- | --- | --- | --- |
| ARS staining | Si-GRB2+PDGFB | 26.85 | 1 | 26.85 | F (1, 8) = 22.24 | P=0.0015 | ** |
|  | Si-GRB2 | 115.2 | 1 | 115.2 | F (1, 8) = 95.38 | P<0.0001 | **** |
|  | PDGFB | 934.2 | 1 | 934.2 | F (1, 8) = 773.8 | P<0.0001 | **** |

Notes: SS, the sum of squares; DF, degree of freedom; MS, mean square.
