# Supplementary material for: Preosteoclast plays a pathogenic role in syndesmophyte formation of ankylosing spondylitis through the secreted PDGFB — GRB2/ERK/RUNX2 pathway
Source: Arthritis Res Ther. 2023 Oct 5;25:194. doi: 10.1186/s13075-023-03142-3 (PMC10552372; doi:10.1186/s13075-023-03142-3)
Supplement: Supplementary file 7 — Additional file 7: Table S7. Results of western blotting of ERK inhibitor and PDGFB treatments analysed by two-way ANOVA. [file 13075_2023_3142_MOESM7_ESM.docx]

Table S7 Results of western blotting of ERK inhibitor and PDGFB treatments analysed by two-way ANOVA.

|  | Effect factors | SS | DF | MS | F (DFn, DFd) | P value | P value summary |
| --- | --- | --- | --- | --- | --- | --- | --- |
| RUNX2 | ERK Inhibitor+PDGFB | 0.1648 | 1 | 0.1648 | F (1, 8) = 37.18 | P=0.0003 | *** |
|  | ERK Inhibitor | 0.6073 | 1 | 0.6073 | F (1, 8) = 137.0 | P<0.0001 | **** |
|  | PDGFB | 0.571 | 1 | 0.571 | F (1, 8) = 128.8 | P<0.0001 | **** |
| ERK | ERK Inhibitor+PDGFB | 0.3031 | 1 | 0.3031 | F (1, 8) = 46.29 | P=0.0001 | *** |
|  | ERK Inhibitor | 0.2641 | 1 | 0.2641 | F (1, 8) = 40.34 | P=0.0002 | *** |
|  | PDGFB | 0.03319 | 1 | 0.03319 | F (1, 8) = 5.069 | P=0.0544 | ns |
| P-ERK | ERK Inhibitor+PDGFB | 0.00584 | 1 | 0.00584 | F (1, 8) = 0.3289 | P=0.5820 | ns |
|  | ERK Inhibitor | 0.2919 | 1 | 0.2919 | F (1, 8) = 16.44 | P=0.0037 | ** |
|  | PDGFB | 0.7406 | 1 | 0.7406 | F (1, 8) = 41.70 | P=0.0002 | *** |
| GRB2 | ERK Inhibitor+PDGFB | 0.00096 | 1 | 0.00096 | F (1, 8) = 0.1637 | P=0.6964 | ns |
|  | ERK Inhibitor | 0.4983 | 1 | 0.4983 | F (1, 8) = 85.07 | P<0.0001 | **** |
|  | PDGFB | 0.7663 | 1 | 0.7663 | F (1, 8) = 130.8 | P<0.0001 | **** |

Notes: SS, the sum of squares; DF, degree of freedom; MS, mean square.
