# Supplementary material for: Preosteoclast plays a pathogenic role in syndesmophyte formation of ankylosing spondylitis through the secreted PDGFB — GRB2/ERK/RUNX2 pathway
Source: Arthritis Res Ther. 2023 Oct 5;25:194. doi: 10.1186/s13075-023-03142-3 (PMC10552372; doi:10.1186/s13075-023-03142-3)
Supplement: Supplementary file 8 — Additional file 8: Table S8. Pathway enrichment of FOB cells according to GO (Biological Process). [file 13075_2023_3142_MOESM8_ESM.docx]

Table S8. Pathway enrichment of FOB cells according to GO (Biological Process).

| NAME_CATEGORY | Adjusted pvalue | Raw pvalue | Fold enrichment | Gene Number |
| --- | --- | --- | --- | --- |
| cardiovascular system development | 7.11E-09 | 2.76E-12 | 5.9 | 23 |
| anatomical structure morphogenesis | 7.11E-09 | 2.50E-12 | 3.39 | 37 |
| circulatory system development | 7.11E-09 | 2.76E-12 | 5.9 | 23 |
| vasculature development | 5.62E-08 | 2.91E-11 | 7.32 | 18 |
| anatomical structure formation involved in morphogenesis | 7.44E-08 | 4.81E-11 | 3.66 | 31 |
| blood vessel development | 1.16E-07 | 9.01E-11 | 7.42 | 17 |
| wound healing | 2.55E-07 | 2.31E-10 | 5.61 | 20 |
| system development | 2.55E-07 | 2.68E-10 | 2.31 | 48 |
| multicellular organismal development | 2.55E-07 | 2.97E-10 | 2.16 | 52 |
| collagen fibril organization | 3.06E-07 | 3.96E-10 | 27.66 | 8 |
| blood vessel morphogenesis | 5.61E-07 | 7.99E-10 | 7.69 | 15 |
| developmental process | 1.57E-06 | 2.44E-09 | 2.02 | 53 |
| angiogenesis | 1.61E-06 | 2.70E-09 | 8.71 | 13 |
| anatomical structure development | 2.88E-06 | 5.22E-09 | 2.1 | 49 |
| regulation of cell adhesion | 3.14E-06 | 6.10E-09 | 8.14 | 13 |
| biological adhesion | 1.33E-05 | 2.92E-08 | 4.23 | 20 |
| cell adhesion | 1.33E-05 | 2.80E-08 | 4.24 | 20 |
| regulation of body fluid levels | 1.89E-05 | 4.41E-08 | 4.92 | 17 |
| multicellular organismal process | 4.32E-05 | 1.06E-07 | 1.74 | 57 |
| coagulation | 4.92E-05 | 1.34E-07 | 5.25 | 15 |
| blood coagulation | 4.92E-05 | 1.34E-07 | 5.25 | 15 |
| hemostasis | 5.22E-05 | 1.48E-07 | 5.21 | 15 |
| locomotion | 6.60E-05 | 1.96E-07 | 3.6 | 21 |
| tissue development | 6.82E-05 | 2.12E-07 | 2.97 | 26 |
| single-multicellular organism process | 8.22E-05 | 2.66E-07 | 1.72 | 56 |
| response to wounding | 9.98E-05 | 3.36E-07 | 3.48 | 21 |
| extracellular structure organization | 1.98E-04 | 7.26E-07 | 7.62 | 10 |
| negative regulation of plasminogen activation | 1.98E-04 | 7.44E-07 | 129.65 | 3 |
| extracellular matrix organization | 1.98E-04 | 6.97E-07 | 7.65 | 10 |
| cellular component morphogenesis | 2.13E-04 | 8.26E-07 | 3.79 | 18 |
| response to steroid hormone stimulus | 2.62E-04 | 1.05E-06 | 6.42 | 11 |
| cell migration | 3.09E-04 | 1.28E-06 | 4.4 | 15 |
| response to external stimulus | 3.62E-04 | 1.54E-06 | 3.17 | 21 |
| response to endogenous stimulus | 5.16E-04 | 2.27E-06 | 3.53 | 18 |
| response to hormone stimulus | 5.27E-04 | 2.39E-06 | 3.92 | 16 |
| response to abiotic stimulus | 5.98E-04 | 2.78E-06 | 3.66 | 17 |
| taxis | 6.59E-04 | 3.24E-06 | 4.37 | 14 |
| chemotaxis | 6.59E-04 | 3.24E-06 | 4.37 | 14 |
| collagen biosynthetic process | 7.06E-04 | 3.69E-06 | 86.44 | 3 |
| cellular component movement | 7.06E-04 | 3.75E-06 | 3.41 | 18 |
| cell motility | 7.06E-04 | 3.84E-06 | 4.02 | 15 |
| localization of cell | 7.06E-04 | 3.84E-06 | 4.02 | 15 |
| platelet activation | 8.26E-04 | 4.59E-06 | 7.14 | 9 |
| regulation of developmental process | 9.98E-04 | 5.68E-06 | 2.73 | 23 |
| regulation of plasminogen activation | 1.11E-03 | 6.43E-06 | 74.09 | 3 |
| cell morphogenesis | 1.16E-03 | 6.90E-06 | 3.61 | 16 |
| cell morphogenesis involved in differentiation | 1.38E-03 | 8.41E-06 | 4.02 | 14 |
| response to organic cyclic compound | 1.41E-03 | 8.74E-06 | 6.59 | 9 |
| response to chemical stimulus | 1.44E-03 | 9.15E-06 | 2.06 | 34 |
| negative regulation of cell adhesion | 1.69E-03 | 1.09E-05 | 11.92 | 6 |
| cell differentiation | 1.90E-03 | 1.26E-05 | 2.15 | 31 |
| tissue morphogenesis | 2.41E-03 | 1.62E-05 | 4.4 | 12 |
| regulation of cell proliferation | 2.57E-03 | 1.76E-05 | 2.81 | 20 |
| growth | 3.04E-03 | 2.12E-05 | 5.21 | 10 |
| regulation of cellular senescence | 3.06E-03 | 2.18E-05 | 51.86 | 3 |
| peptide cross-linking | 3.06E-03 | 2.22E-05 | 23.84 | 4 |
| neurogenesis | 3.25E-03 | 2.40E-05 | 2.86 | 19 |
| response to corticosteroid stimulus | 3.40E-03 | 2.55E-05 | 8.07 | 7 |
| regulation of blood coagulation | 3.42E-03 | 2.66E-05 | 14.17 | 5 |
| regulation of hemostasis | 3.42E-03 | 2.66E-05 | 14.17 | 5 |
| cellular developmental process | 3.50E-03 | 2.76E-05 | 2.07 | 31 |
| response to gravity | 3.65E-03 | 2.98E-05 | 47.15 | 3 |
| response to organic nitrogen | 3.65E-03 | 3.02E-05 | 7.86 | 7 |
| regulation of multicellular organismal process | 3.65E-03 | 2.98E-05 | 2.34 | 25 |
| leukocyte migration | 3.82E-03 | 3.28E-05 | 6.46 | 8 |
| oncogene-induced senescence | 3.82E-03 | 3.31E-05 | 172.87 | 2 |
| gland development | 3.82E-03 | 3.30E-05 | 5.58 | 9 |
| positive regulation of developmental process | 3.91E-03 | 3.44E-05 | 3.54 | 14 |
| negative regulation of protein processing | 4.44E-03 | 3.96E-05 | 43.22 | 3 |
| axon guidance | 4.44E-03 | 4.12E-05 | 4.82 | 10 |
| response to cAMP | 4.44E-03 | 4.20E-05 | 12.9 | 5 |
| regulation of coagulation | 4.44E-03 | 4.20E-05 | 12.9 | 5 |
| generation of neurons | 4.44E-03 | 4.08E-05 | 2.86 | 18 |
| cellular response to stimulus | 4.51E-03 | 4.32E-05 | 1.65 | 46 |
| positive regulation of gene expression | 4.74E-03 | 4.60E-05 | 2.83 | 18 |
| positive regulation of macromolecule metabolic process | 5.15E-03 | 5.06E-05 | 2.27 | 25 |
| regulation of biological quality | 5.33E-03 | 5.30E-05 | 2.08 | 29 |
| regulation of response to stimulus | 5.39E-03 | 5.43E-05 | 2.03 | 30 |
| negative regulation of blood coagulation | 5.48E-03 | 5.96E-05 | 18.69 | 4 |
| positive regulation of cell cycle | 5.48E-03 | 5.61E-05 | 8.94 | 6 |
| response to vitamin | 5.48E-03 | 5.68E-05 | 7.12 | 7 |
| regulation of multicellular organismal development | 5.48E-03 | 5.82E-05 | 2.78 | 18 |
| negative regulation of hemostasis | 5.48E-03 | 5.96E-05 | 18.69 | 4 |
| skin development | 5.48E-03 | 5.94E-05 | 12.01 | 5 |
| negative regulation of multicellular organismal process | 5.79E-03 | 6.36E-05 | 4.57 | 10 |
| skeletal system development | 5.98E-03 | 6.65E-05 | 4.55 | 10 |
| enzyme linked receptor protein signaling pathway | 6.30E-03 | 7.08E-05 | 3.32 | 14 |
| single-organism process | 6.33E-03 | 7.20E-05 | 1.4 | 62 |
| organ development | 6.45E-03 | 7.43E-05 | 2 | 30 |
| response to organic substance | 6.93E-03 | 8.25E-05 | 2.25 | 24 |
| response to extracellular stimulus | 6.93E-03 | 8.25E-05 | 4.43 | 10 |
| epidermis morphogenesis | 6.93E-03 | 8.14E-05 | 17.29 | 4 |
| positive regulation of cell adhesion | 7.16E-03 | 8.89E-05 | 8.23 | 6 |
| negative regulation of coagulation | 7.16E-03 | 8.98E-05 | 16.87 | 4 |
| tissue regeneration | 7.16E-03 | 8.98E-05 | 16.87 | 4 |
| regulation of wound healing | 7.16E-03 | 8.73E-05 | 11.08 | 5 |
| response to stimulus | 7.16E-03 | 8.71E-05 | 1.46 | 56 |
| regulation of smooth muscle cell proliferation | 7.32E-03 | 9.27E-05 | 10.94 | 5 |
| apoptotic cell clearance | 7.66E-03 | 9.91E-05 | 32.41 | 3 |
| senescence-associated heterochromatin focus assembly | 7.66E-03 | 9.89E-05 | 115.25 | 2 |
| platelet degranulation | 8.48E-03 | 1.11E-04 | 10.54 | 5 |
| regulation of cell migration | 8.54E-03 | 1.13E-04 | 4.27 | 10 |
| developmental growth | 9.46E-03 | 1.26E-04 | 6.27 | 7 |
| response to mechanical stimulus | 9.97E-03 | 1.35E-04 | 7.63 | 6 |
| response to oxygen levels | 9.97E-03 | 1.35E-04 | 5.28 | 8 |
| fibrinolysis | 1.03E-02 | 1.43E-04 | 28.81 | 3 |
| regulation of cell aging | 1.03E-02 | 1.43E-04 | 28.81 | 3 |
| positive regulation of signaling | 1.08E-02 | 1.51E-04 | 3.09 | 14 |
| positive regulation of cell communication | 1.08E-02 | 1.53E-04 | 3.08 | 14 |
| neuron projection morphogenesis | 1.13E-02 | 1.60E-04 | 3.74 | 11 |
| response to glucocorticoid stimulus | 1.15E-02 | 1.65E-04 | 7.36 | 6 |
| positive regulation of metabolic process | 1.17E-02 | 1.71E-04 | 2.11 | 25 |
| regulation of cell motility | 1.17E-02 | 1.70E-04 | 4.06 | 10 |
| positive regulation of cellular senescence | 1.34E-02 | 1.97E-04 | 86.44 | 2 |
| cell surface receptor signaling pathway | 1.34E-02 | 1.99E-04 | 2.04 | 26 |
| negative regulation of biological process | 1.34E-02 | 2.01E-04 | 1.81 | 33 |
| regulation of signal transduction | 1.45E-02 | 2.20E-04 | 2.17 | 23 |
| regulation of signaling | 1.50E-02 | 2.28E-04 | 2.07 | 25 |
| regulation of cell communication | 1.56E-02 | 2.41E-04 | 2.06 | 25 |
| response to estrogen stimulus | 1.65E-02 | 2.57E-04 | 6.78 | 6 |
| positive regulation of biological process | 1.65E-02 | 2.58E-04 | 1.72 | 36 |
| regulation of cellular response to growth factor stimulus | 1.68E-02 | 2.65E-04 | 12.81 | 4 |
| nervous system development | 1.69E-02 | 2.68E-04 | 2.19 | 22 |
| regulation of angiogenesis | 1.72E-02 | 2.76E-04 | 6.69 | 6 |
| response to inorganic substance | 1.78E-02 | 2.88E-04 | 4.19 | 9 |
| regulation of locomotion | 1.80E-02 | 2.94E-04 | 3.79 | 10 |
| positive regulation of signal transduction | 1.93E-02 | 3.16E-04 | 3.03 | 13 |
| positive regulation of macromolecule biosynthetic process | 2.03E-02 | 3.35E-04 | 2.51 | 17 |
| regulation of cellular component movement | 2.06E-02 | 3.43E-04 | 3.72 | 10 |
| positive regulation of apoptotic process | 2.15E-02 | 3.61E-04 | 3.69 | 10 |
| axonogenesis | 2.21E-02 | 3.74E-04 | 3.68 | 10 |
| positive regulation of response to external stimulus | 2.26E-02 | 3.86E-04 | 6.29 | 6 |
| response to progesterone stimulus | 2.26E-02 | 3.92E-04 | 20.74 | 3 |
| response to vitamin D | 2.26E-02 | 3.92E-04 | 20.74 | 3 |
| positive regulation of programmed cell death | 2.33E-02 | 4.06E-04 | 3.64 | 10 |
| regulation of vasculature development | 2.34E-02 | 4.11E-04 | 6.21 | 6 |
| regulation of system process | 2.35E-02 | 4.20E-04 | 3.62 | 10 |
| response to stress | 2.35E-02 | 4.19E-04 | 1.79 | 31 |
| positive regulation of cellular process | 2.47E-02 | 4.45E-04 | 1.74 | 33 |
| positive regulation of response to stimulus | 2.56E-02 | 4.63E-04 | 2.44 | 17 |
| heterochromatin organization | 2.64E-02 | 4.89E-04 | 57.62 | 2 |
| positive regulation of cell aging | 2.64E-02 | 4.89E-04 | 57.62 | 2 |
| heterochromatin assembly | 2.64E-02 | 4.89E-04 | 57.62 | 2 |
| positive regulation of cell death | 2.69E-02 | 5.02E-04 | 3.54 | 10 |
| negative regulation of protein metabolic process | 2.85E-02 | 5.35E-04 | 3.51 | 10 |
| organ morphogenesis | 2.87E-02 | 5.41E-04 | 2.87 | 13 |
| response to reactive oxygen species | 2.94E-02 | 5.58E-04 | 7.45 | 5 |
| cell development | 2.96E-02 | 5.70E-04 | 2.4 | 17 |
| neuron projection development | 2.96E-02 | 5.67E-04 | 3.23 | 11 |
| response to hypoxia | 2.96E-02 | 5.79E-04 | 4.88 | 7 |
| connective tissue development | 2.96E-02 | 5.76E-04 | 5.83 | 6 |
| cell morphogenesis involved in neuron differentiation | 2.99E-02 | 5.88E-04 | 3.47 | 10 |
| response to decreased oxygen levels | 3.07E-02 | 6.07E-04 | 4.84 | 7 |
| regulation of response to external stimulus | 3.21E-02 | 6.38E-04 | 3.76 | 9 |
| skeletal system morphogenesis | 3.23E-02 | 6.48E-04 | 5.7 | 6 |
| regeneration | 3.23E-02 | 6.52E-04 | 7.2 | 5 |
| transmembrane receptor protein serine/threonine kinase signaling pathway | 3.26E-02 | 6.67E-04 | 5.67 | 6 |
| response to nutrient | 3.26E-02 | 6.67E-04 | 4.76 | 7 |
| menstrual cycle phase | 3.29E-02 | 6.82E-04 | 49.39 | 2 |
| cell projection morphogenesis | 3.29E-02 | 6.80E-04 | 3.16 | 11 |
| negative regulation of response to stimulus | 3.49E-02 | 7.35E-04 | 2.77 | 13 |
| response to growth factor stimulus | 3.49E-02 | 7.31E-04 | 4.69 | 7 |
| aging | 3.49E-02 | 7.26E-04 | 5.58 | 6 |
| negative regulation of cell-substrate adhesion | 3.52E-02 | 7.47E-04 | 16.73 | 3 |
| response to amino acid stimulus | 3.73E-02 | 7.96E-04 | 9.6 | 4 |
| cell part morphogenesis | 3.77E-02 | 8.10E-04 | 3.09 | 11 |
| regulation of smooth muscle cell migration | 3.80E-02 | 8.21E-04 | 16.21 | 3 |
| cell communication | 3.81E-02 | 8.28E-04 | 1.56 | 40 |
| protein phosphorylation | 3.86E-02 | 8.43E-04 | 3.08 | 11 |
| signal transduction | 4.00E-02 | 8.80E-04 | 1.62 | 36 |
| bone trabecula formation | 4.07E-02 | 9.05E-04 | 43.22 | 2 |
| response to L-ascorbic acid | 4.07E-02 | 9.05E-04 | 43.22 | 2 |
| positive regulation of chemotaxis | 4.36E-02 | 9.75E-04 | 9.1 | 4 |
| protein metabolic process | 4.39E-02 | 9.88E-04 | 1.69 | 32 |
| negative regulation of cell migration | 4.74E-02 | 1.07E-03 | 6.45 | 5 |
| neuron differentiation | 4.77E-02 | 1.09E-03 | 2.66 | 13 |
