# Supplementary material for: Preosteoclast plays a pathogenic role in syndesmophyte formation of ankylosing spondylitis through the secreted PDGFB — GRB2/ERK/RUNX2 pathway
Source: Arthritis Res Ther. 2023 Oct 5;25:194. doi: 10.1186/s13075-023-03142-3 (PMC10552372; doi:10.1186/s13075-023-03142-3)
Supplement: Supplementary file 9 — Additional file 9: Table S9. Pathway enrichment of FOB cells according to KEGG. [file 13075_2023_3142_MOESM9_ESM.docx]

Table S9. Pathway enrichment of FOB cells according to KEGG.

| NAME_CATEGORY | Adjusted pvalue | Raw pvalue | Fold enrichment | Gene Number |
| --- | --- | --- | --- | --- |
| Focal adhesion | 4.07E-05 | 1.95E-07 | 7.27 | 11 |
| ECM-receptor interaction | 7.67E-05 | 7.36E-07 | 10.43 | 8 |
| Amoebiasis | 1.01E-04 | 1.45E-06 | 9.55 | 8 |
| Protein digestion and absorption | 1.82E-03 | 3.48E-05 | 9.49 | 6 |
| Malaria | 2.35E-01 | 7.90E-03 | 7.28 | 3 |
| Aminoacyl-tRNA biosynthesis | 2.35E-01 | 5.83E-03 | 8.12 | 3 |
| Hematopoietic cell lineage | 2.35E-01 | 7.47E-03 | 5.12 | 4 |
| p53 signaling pathway | 3.18E-01 | 1.22E-02 | 6.21 | 3 |
| Melanoma | 3.18E-01 | 1.37E-02 | 5.95 | 3 |
| Complement and coagulation cascades | 4.62E-01 | 2.21E-02 | 4.97 | 3 |
| Dilated cardiomyopathy | 4.64E-01 | 3.12E-02 | 4.35 | 3 |
| Hypertrophic cardiomyopathy (HCM) | 4.64E-01 | 2.57E-02 | 4.69 | 3 |
| TGF-beta signaling pathway | 4.64E-01 | 2.80E-02 | 4.54 | 3 |
| Pathways in cancer | 4.64E-01 | 2.94E-02 | 2.54 | 6 |
| Bladder cancer | 5.16E-01 | 3.71E-02 | 6.55 | 2 |
| Chagas disease (American trypanosomiasis) | 6.23E-01 | 4.78E-02 | 3.67 | 3 |
| MAPK signaling pathway | 6.73E-01 | 5.49E-02 | 2.41 | 5 |
| Axon guidance | 7.33E-01 | 6.67E-02 | 3.2 | 3 |
| Regulation of actin cytoskeleton | 7.33E-01 | 6.36E-02 | 2.64 | 4 |
| Colorectal cancer | 7.45E-01 | 7.14E-02 | 4.54 | 2 |
| Glioma | 7.70E-01 | 7.75E-02 | 4.33 | 2 |
| Pancreatic cancer | 7.98E-01 | 8.80E-02 | 4.02 | 2 |
| Bacterial invasion of epithelial cells | 7.98E-01 | 8.80E-02 | 4.02 | 2 |
| Chronic myeloid leukemia | 8.07E-01 | 9.45E-02 | 3.86 | 2 |
| Arrhythmogenic right ventricular cardiomyopathy (ARVC) | 8.07E-01 | 9.67E-02 | 3.81 | 2 |
| Wnt signaling pathway | 8.25E-01 | 1.03E-01 | 2.66 | 3 |
| Osteoclast differentiation | 9.10E-01 | 1.18E-01 | 2.5 | 3 |
| Prostate cancer | 9.44E-01 | 1.29E-01 | 3.2 | 2 |
| Pertussis | 9.44E-01 | 1.41E-01 | 3.03 | 2 |
| Cytokine-cytokine receptor interaction | 9.44E-01 | 1.49E-01 | 1.94 | 4 |
| Apoptosis | 9.44E-01 | 1.41E-01 | 3.03 | 2 |
| Small cell lung cancer | 9.44E-01 | 1.34E-01 | 3.13 | 2 |
| Transcriptional misregulation in cancer | 9.44E-01 | 1.45E-01 | 2.27 | 3 |
| Gap junction | 9.56E-01 | 1.56E-01 | 2.84 | 2 |
| Measles | 1.00E+00 | 2.99E-01 | 1.83 | 2 |
| Type I diabetes mellitus | 1.00E+00 | 3.26E-01 | 1.72 | 2 |
| HTLV-I infection | 1.00E+00 | 2.95E-01 | 1.46 | 4 |
| Rheumatoid arthritis | 1.00E+00 | 3.83E-01 | 1.51 | 2 |
| Alzheimer's disease | 1.00E+00 | 3.80E-01 | 1.52 | 2 |
| Protein processing in endoplasmic reticulum | 1.00E+00 | 3.90E-01 | 1.49 | 2 |
| Cell cycle | 1.00E+00 | 2.25E-01 | 2.23 | 2 |
| Leishmaniasis | 1.00E+00 | 2.94E-01 | 1.85 | 2 |
| Tuberculosis | 1.00E+00 | 5.56E-01 | 1.08 | 2 |
| Cell adhesion molecules (CAMs) | 1.00E+00 | 5.26E-01 | 1.14 | 2 |
| Jak-STAT signaling pathway | 1.00E+00 | 3.12E-01 | 1.77 | 2 |
| Phagosome | 1.00E+00 | 3.44E-01 | 1.45 | 3 |
| Metabolic pathways | 1.00E+00 | 9.54E-01 | 0.57 | 5 |
