# Supplementary material for: Preosteoclast plays a pathogenic role in syndesmophyte formation of ankylosing spondylitis through the secreted PDGFB — GRB2/ERK/RUNX2 pathway
Source: Arthritis Res Ther. 2023 Oct 5;25:194. doi: 10.1186/s13075-023-03142-3 (PMC10552372; doi:10.1186/s13075-023-03142-3)
Supplement: Supplementary file 10 — Additional file 10: Table S10. Results of western blotting of si-RUNX2 and PDGFB treatments analysed by two-way ANOVA. [file 13075_2023_3142_MOESM10_ESM.docx]

Table S10 Results of western blotting of si-RUNX2 and PDGFB treatments analysed by two-way ANOVA.

|  | Effect factors | SS | DF | MS | F (DFn, DFd) | P value | P value summary |
| --- | --- | --- | --- | --- | --- | --- | --- |
| COL1 | Si-RUNX2+PDGFB | 0.00032 | 1 | 0.00032 | F (1, 8) = 0.005778 | P=0.9413 | ns |
|  | Si-RUNX2 | 0.1859 | 1 | 0.1859 | F (1, 8) = 3.319 | P=0.1060 | ns |
|  | PDGFB | 0.7531 | 1 | 0.7531 | F (1, 8) = 13.44 | P=0.0063 | ** |
| COL3 | Si-RUNX2+PDGFB | 0.5447 | 1 | 0.5447 | F (1, 8) = 17.71 | P=0.0030 | ** |
|  | Si-RUNX2 | 0.2279 | 1 | 0.2279 | F (1, 8) = 7.412 | P=0.0261 | * |
|  | PDGFB | 0.333 | 1 | 0.333 | F (1, 8) = 10.83 | P=0.0110 | * |
| RUNX2 | Si-RUNX2+PDGFB | 0.02115 | 1 | 0.02115 | F (1, 8) = 7.793 | P=0.0235 | * |
|  | Si-RUNX2 | 0.01689 | 1 | 0.01689 | F (1, 8) = 6.222 | P=0.0373 | * |
|  | PDGFB | 0.03538 | 1 | 0.03538 | F (1, 8) = 13.04 | P=0.0069 | ** |

Notes: SS, the sum of squares; DF, degree of freedom; MS, mean square.
