# Supplementary figures and images for: Preosteoclast plays a pathogenic role in syndesmophyte formation of ankylosing spondylitis through the secreted PDGFB — GRB2/ERK/RUNX2 pathway
Source: Arthritis Res Ther. 2023 Oct 5;25:194. doi: 10.1186/s13075-023-03142-3 (PMC10552372; doi:10.1186/s13075-023-03142-3)

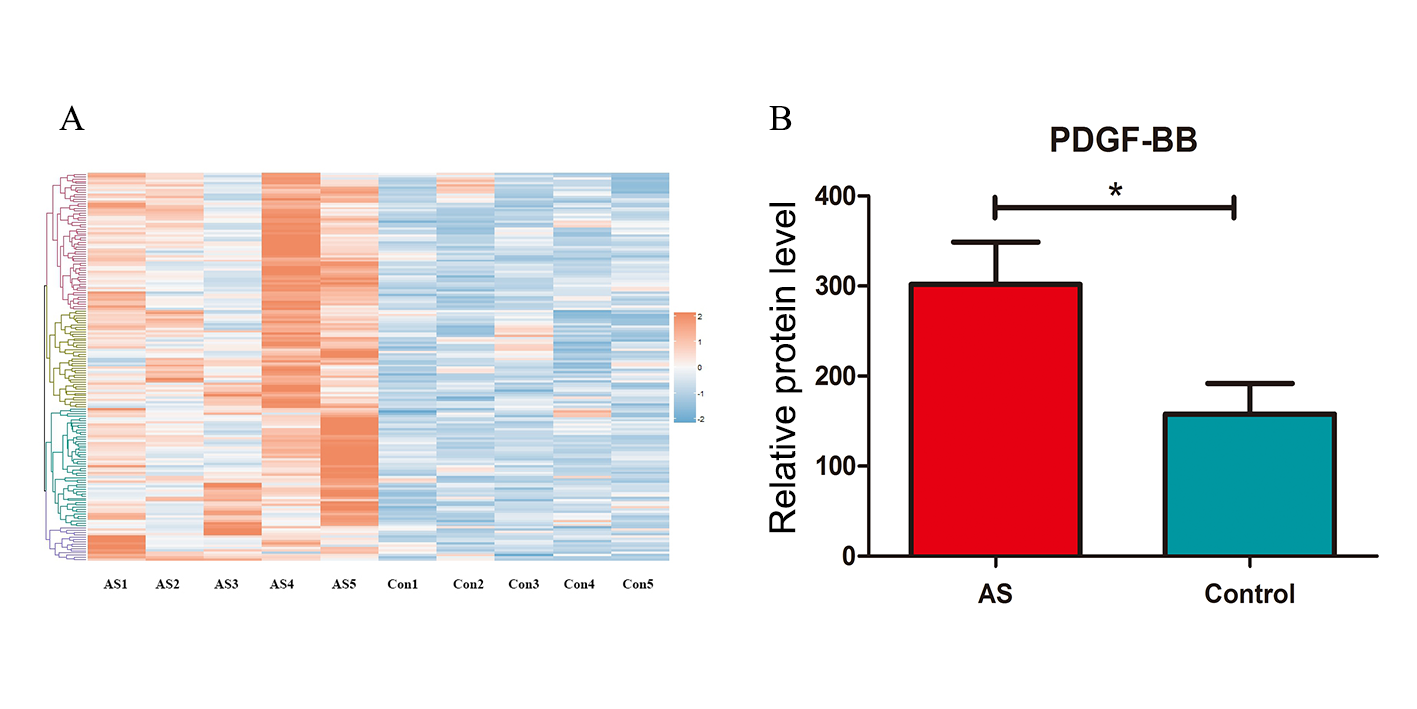

Supplement: Supplementary file 11 — Additional file 11: Figure S1. A, Heatmap plot of AS and controls. B, PDGFB expression in AS patients and controls. The relative protein levels were compared by unpaired sample t-tests. * P < 0.05. [file 13075_2023_3142_MOESM11_ESM.tif]

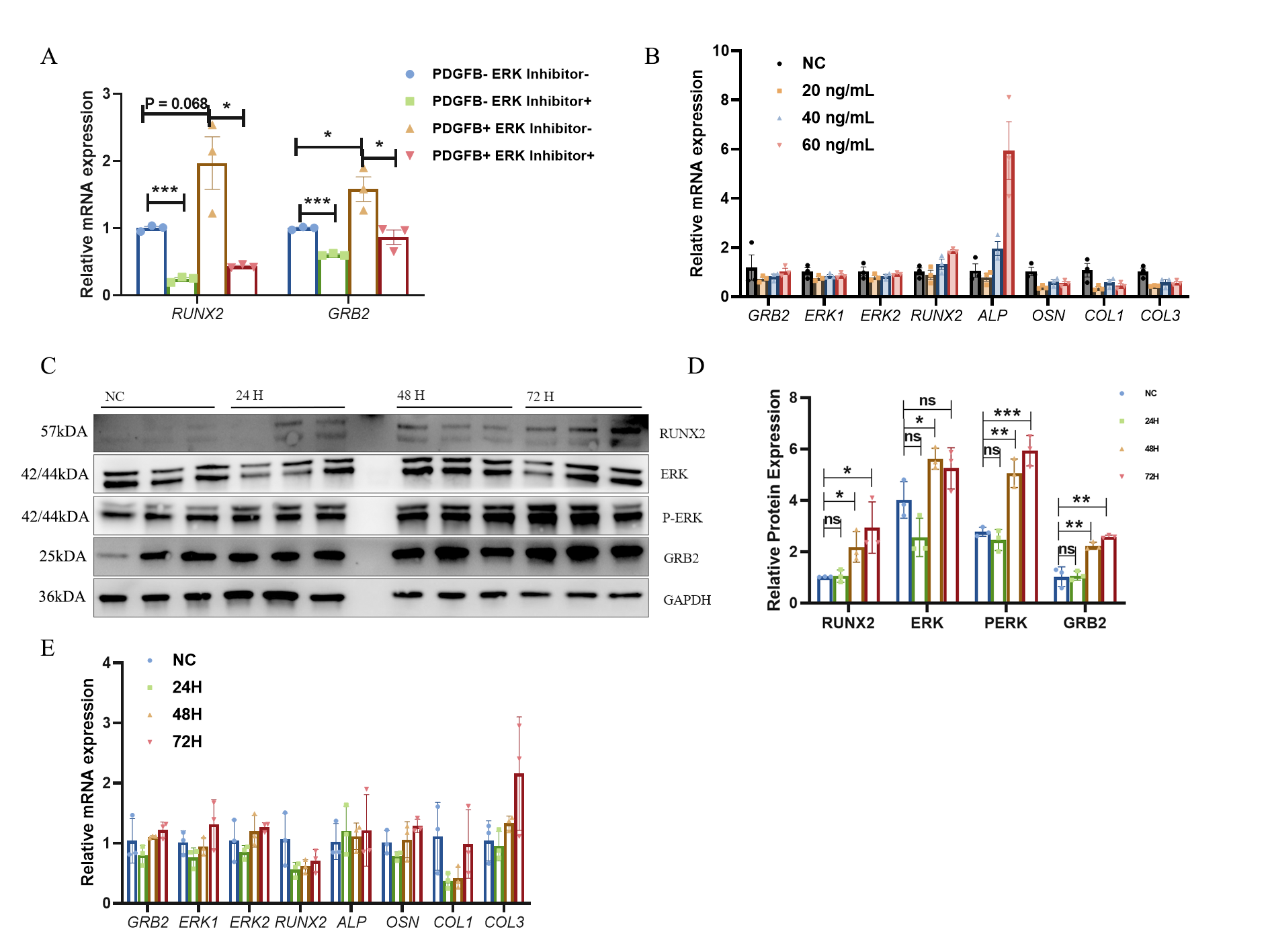

Supplement: Supplementary file 12 — Additional file 12: Figure S2. PDGFB pathway regulation in ADSCs. A, The mRNA expression of GRB2 and RUNX2 with/without the treatments of PDGFB and ERK inhibitor. B, The mRNA expression of several key genes in GRB2-pERK-RUNX2 axis and osteogenesis-related genes, in dose-dependent groups. C-D, Western blot of several key molecules in the axis with the treatment of PDGFB in time-dependent groups. E, The mRNA expression of several key genes in the axis and osteogenesis-related genes, in time-dependent groups. [file 13075_2023_3142_MOESM12_ESM.tif]

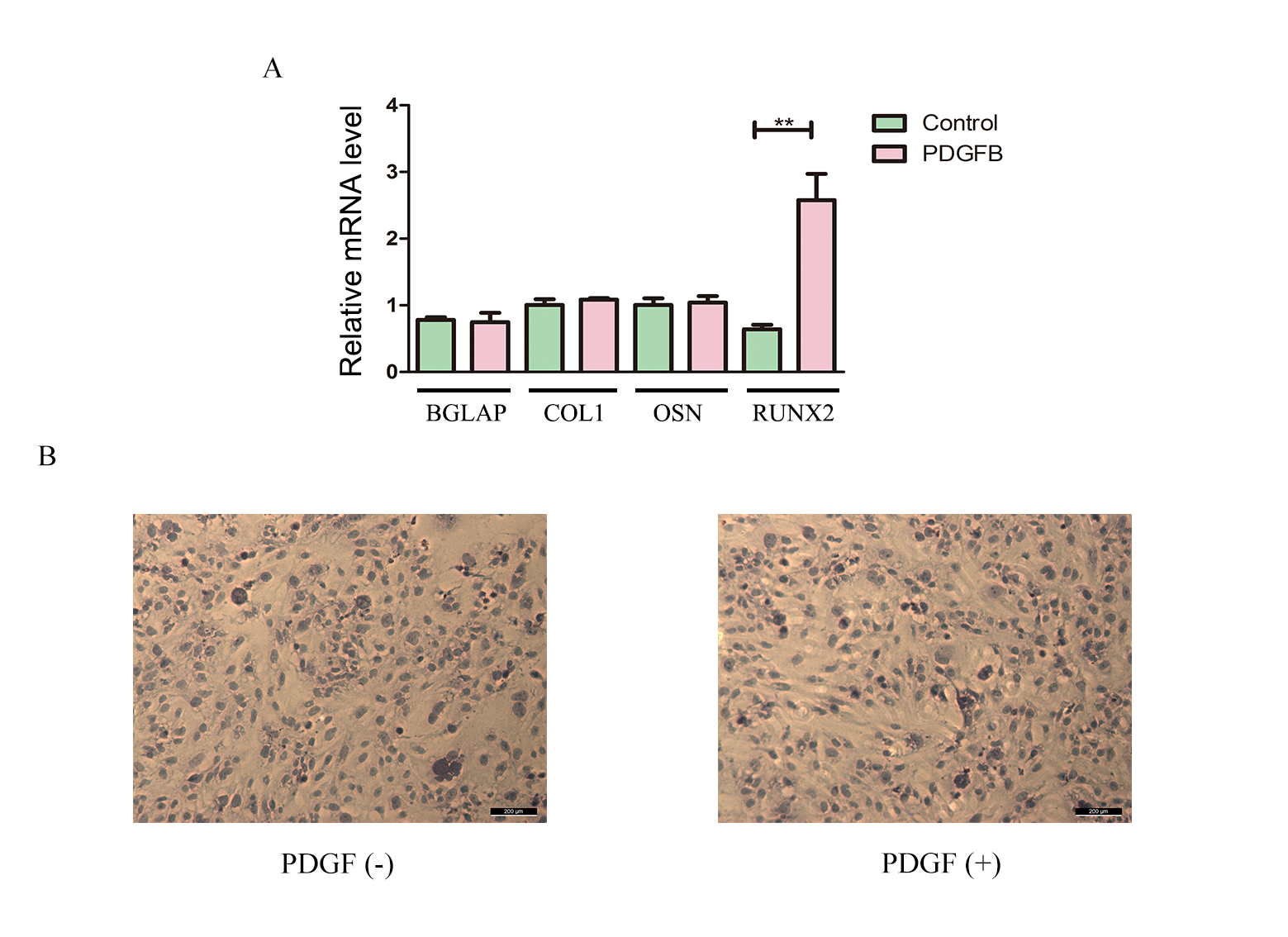

Supplement: Supplementary file 13 — Additional file 13: Figure S3. A, The mRNA expression of osteogenic-related genes in FOB1.19 cells. B, ALP staining of FOB1.19 in the two groups. The gene expression levels were compared by unpaired sample t-tests. ** P < 0.01. [file 13075_2023_3142_MOESM13_ESM.tif]

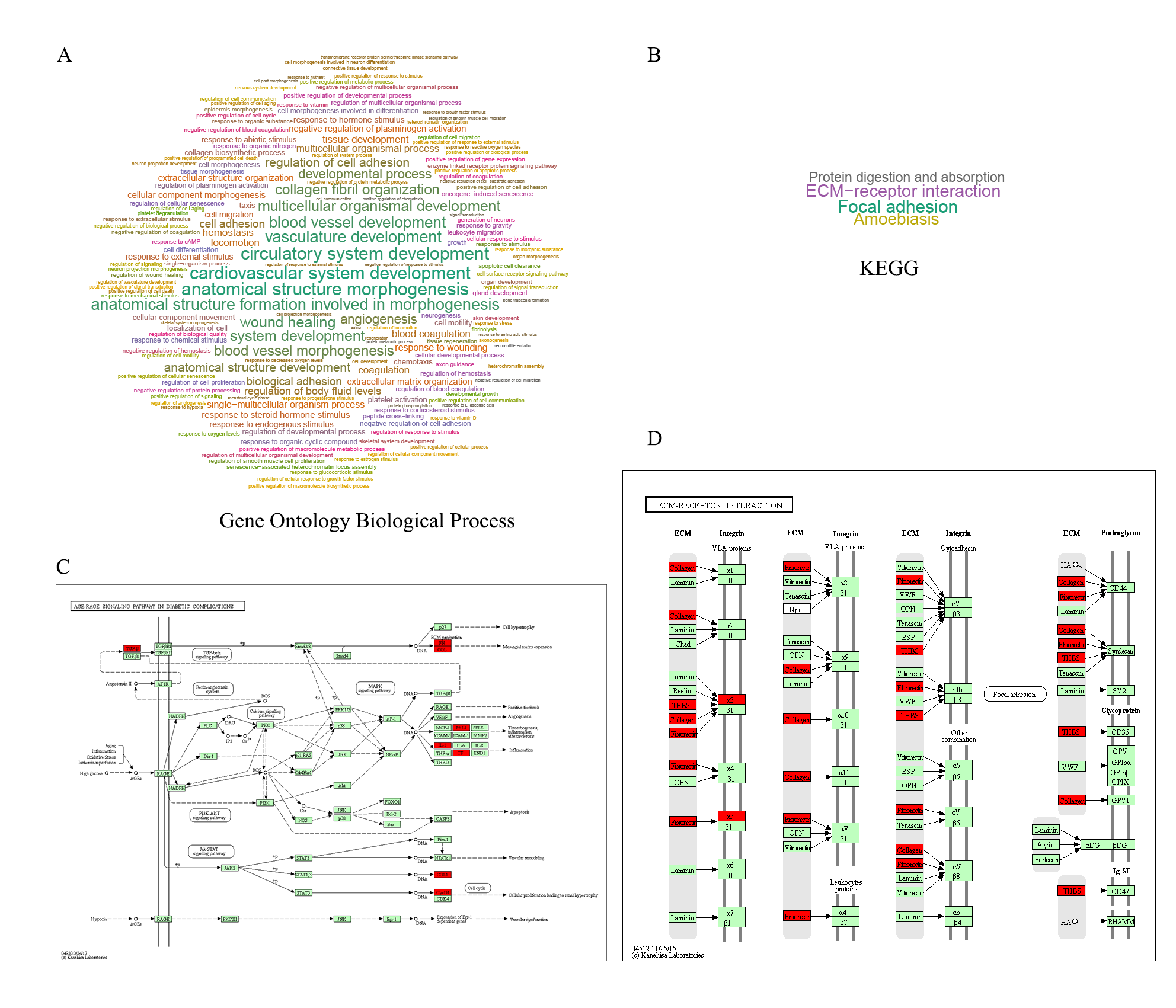

Supplement: Supplementary file 14 — Additional file 14: Figure S4. A, GO enrichment analysis of RNA sequencing in FOB1.19 cells. B, KEGG enrichment analysis of RNA sequencing in FOB1.19 cells. C, Enriched AGE-RAGE pathway in FOB1.19 cells. D, Enriched ECM-receptor interaction pathway in FOB1.19 cells. [file 13075_2023_3142_MOESM14_ESM.tif]

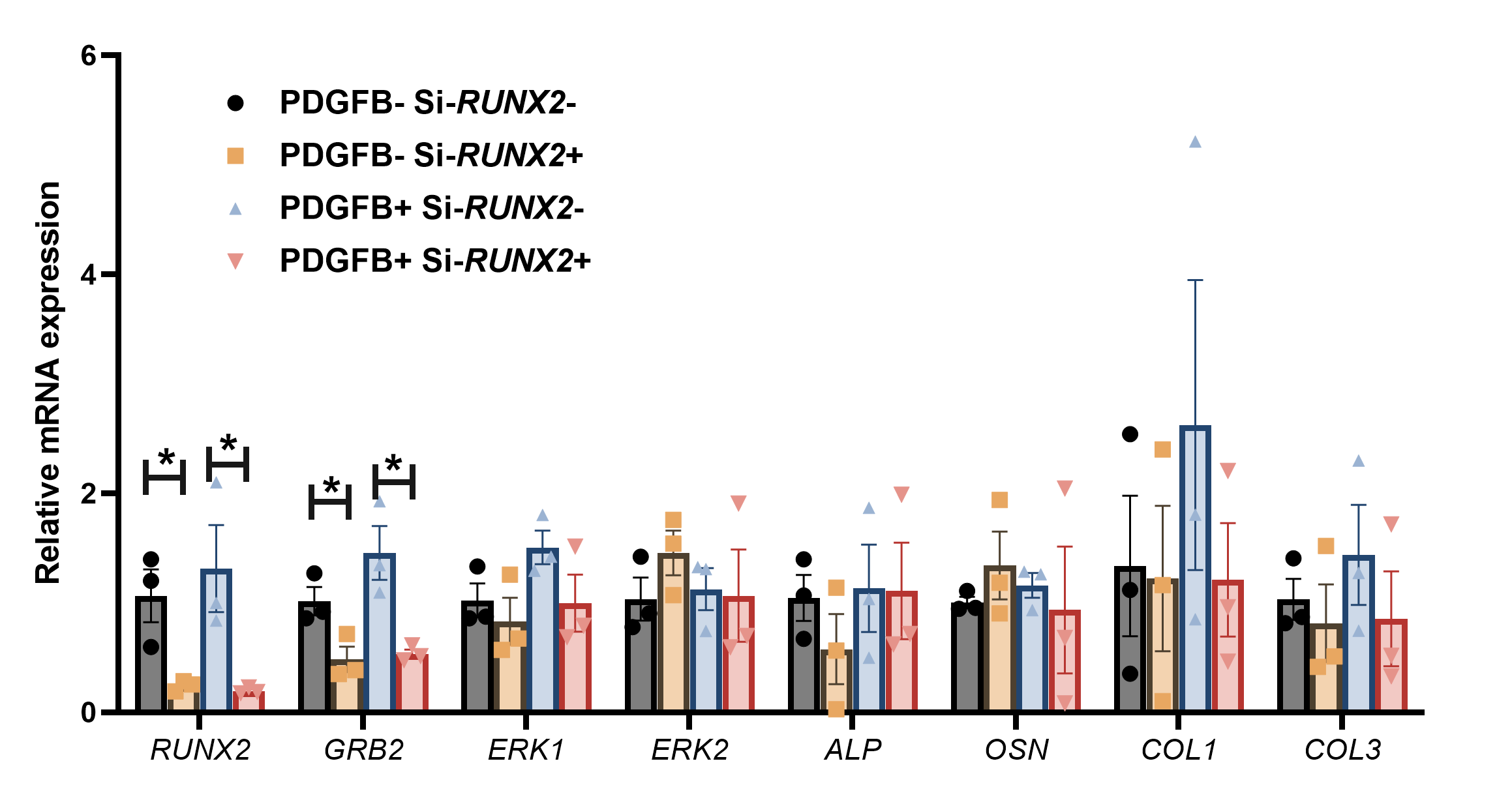

Supplement: Supplementary file 15 — Additional file 15: Figure S5. The mRNA expression of several key genes in the GRB2-pERK-RUNX2 axis and several osteogenesis related genes, with/without the treatments of PDGFB and si-RUNX2. [file 13075_2023_3142_MOESM15_ESM.tif]
